# Supplementary material for: Estimating direct and indirect genetic effects on offspring phenotypes using genome-wide summary results data
Source: Nat Commun. 2021 Sep 14;12:5420. doi: 10.1038/s41467-021-25723-z (PMC8440517; doi:10.1038/s41467-021-25723-z)
Supplement: Supplementary file 1 — Supplementary Information [file 41467_2021_25723_MOESM1_ESM.pdf]

Supplementary Figure 1: Manhattan plot and quantile-quantile (Q-Q) plot for the offspring specific effect estimated using the structural equation model (SEM) with summary statistics and no sample overlap between the GWAS of own and offspring birth weight. We calculated a Wald P-value for the offspring genetic effects from the SEM using their effect size estimates and standard errors. The two-sided association P-value, on the –log10 scale, obtained from the SEM for each of the SNPs (y-axis) was plotted against the genomic position (NCBI Build 37; x-axis). Association signals that reached genome-wide significance ($P < 5x10^{-8}$) are shown in red if they are novel and turquoise if they have been previously reported[3]. In the Q-Q plots, the black dots represent observed P-values and the grey line represents expected P-values under the null distribution. The green dots represent observed P-values after excluding the previously identified signals[3]. All P-values are two sided and not adjusted for multiple comparisons.

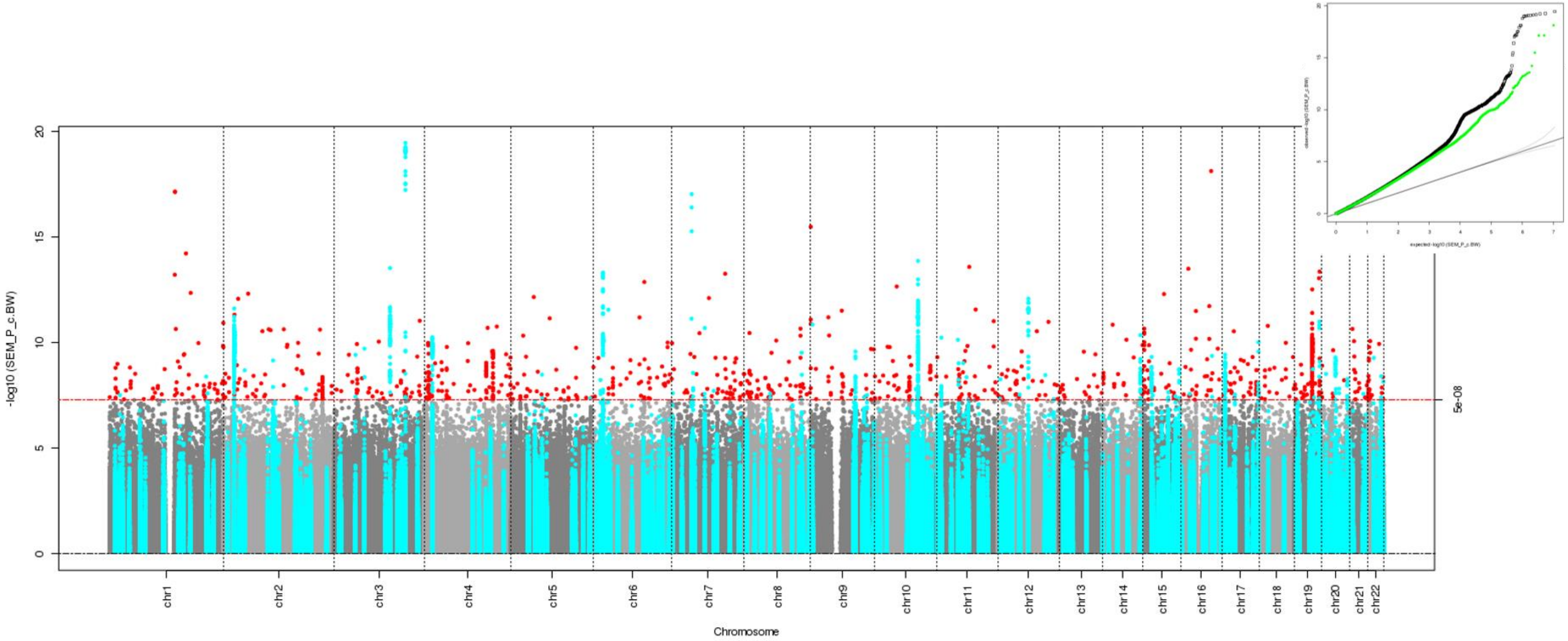

Supplementary Figure 2: Manhattan plot and quantile-quantile (Q-Q) plot for the offspring specific effect estimated using the linear approximation of the structural equation model (SEM) and no sample overlap between the GWAS of own and offspring birth weight. We calculated a Z test for the offspring genetic effects using their effect size estimates and standard errors. The two-sided association P-value, on the –log10 scale, obtained from the linear approximation of the SEM for each of the SNPs (y-axis) was plotted against the genomic position (NCBI Build 37; x-axis). Association signals that reached genome-wide significance ($P < 5x10^{-8}$) are shown in red if they are novel and turquoise if they have been previously reported[3]. In the Q-Q plots, the black dots represent observed P-values and the grey line represents expected P-values under the null distribution. The green dots represent observed P-values after excluding the previously identified signals[3]. All P-values are two sided and not adjusted for multiple comparisons.

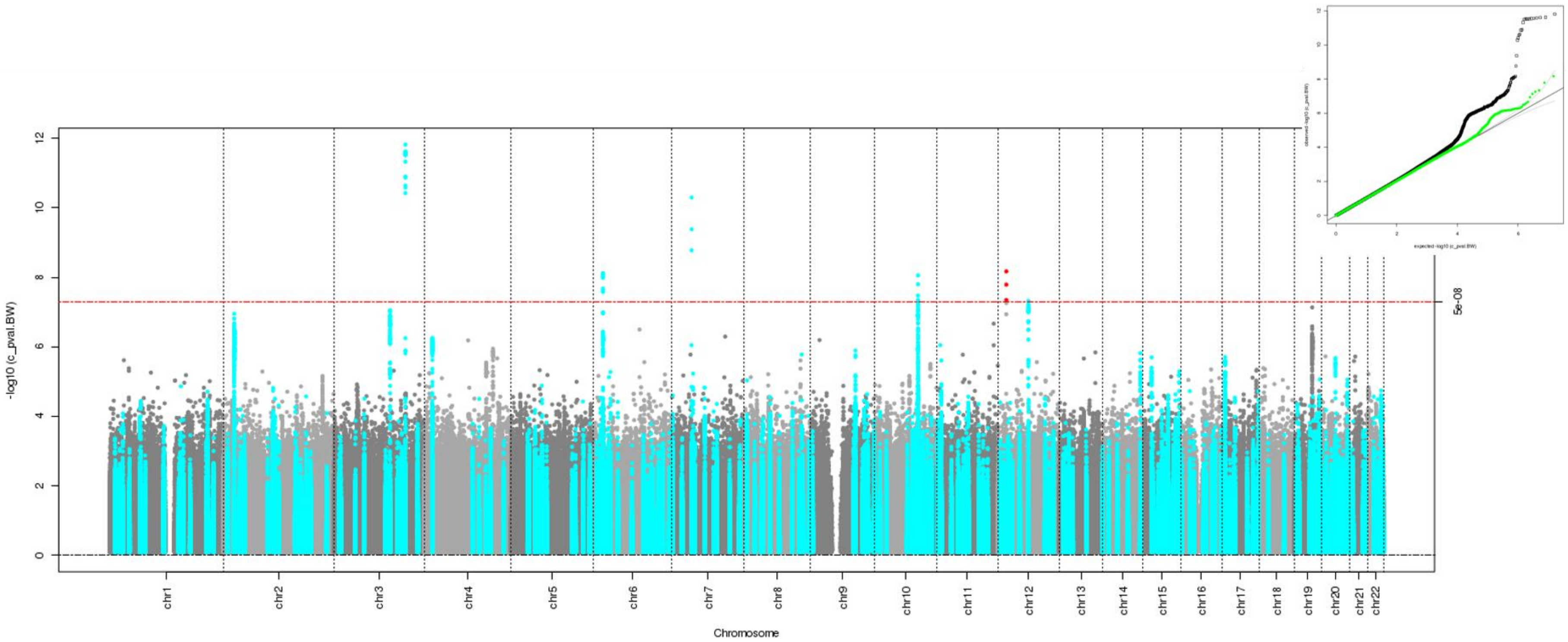

Supplementary Figure 3: Manhattan plot and quantile-quantile (Q-Q) plot for the offspring specific effect estimated using MTAG[4] and no sample overlap between the GWAS of own and offspring birth weight. MTAG calculates a Z test using effect size estimates and standard errors. The two-sided association P-value, on the –log10 scale, obtained from MTAG for each of the SNPs (y-axis) was plotted against the genomic position (NCBI Build 37; x-axis). Association signals that reached genome-wide significance ($P < 5x10^{-8}$) are shown in red if they are novel and turquoise if they have been previously reported[3]. In the Q-Q plots, the black dots represent observed P-values and the grey line represents expected P-values under the null distribution. The green dots represent observed P-values after excluding the previously identified signals[3]. All P-values are two sided and not adjusted for multiple comparisons.

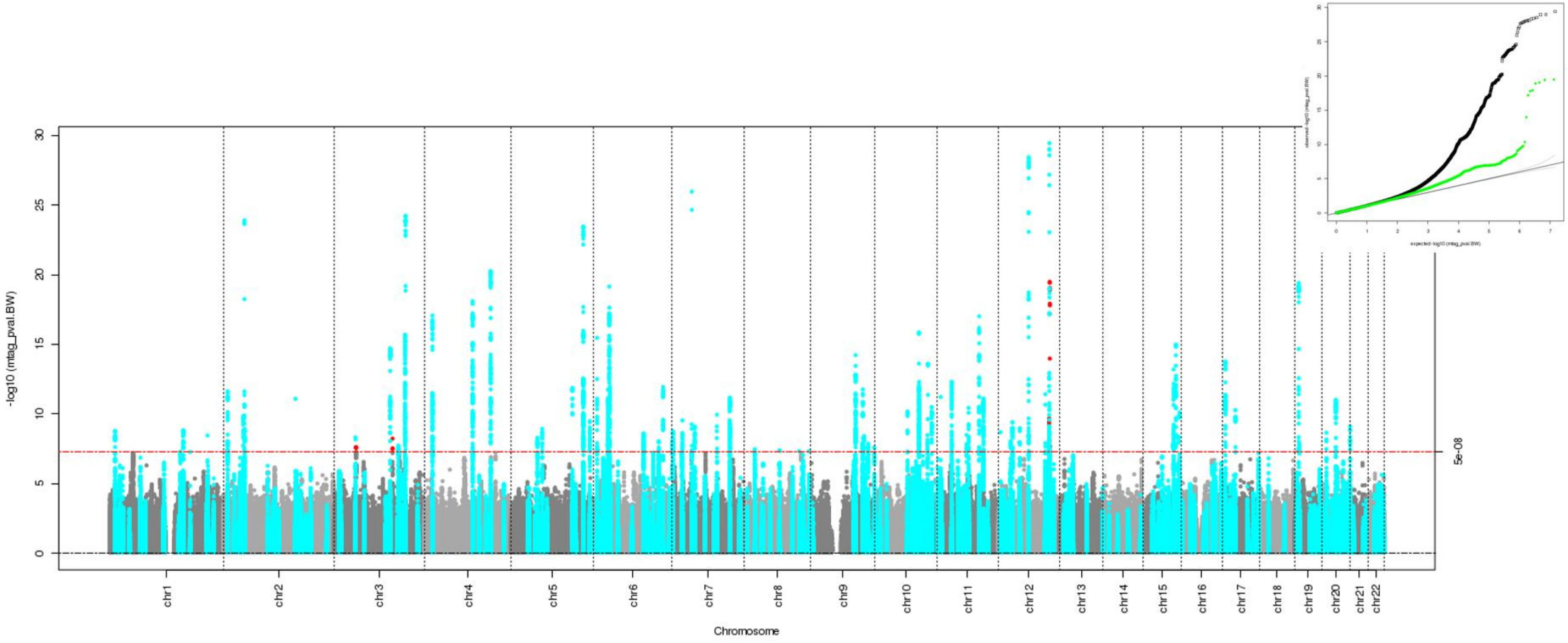

Supplementary Figure 4: Manhattan plot and quantile-quantile (Q-Q) plot for the offspring specific effect estimated using mtCOJO[5] and no sample overlap between the GWAS of own and offspring birth weight. mtCOJO calculates a chi-square test using effect size estimates and standard errors. The two-sided association P-value, on the –log10 scale, obtained from mtCOJO for each of the SNPs (y-axis) was plotted against the genomic position (NCBI Build 37; x-axis). Association signals that reached genome-wide significance ($P < 5x10^{-8}$) are shown in red if they are novel and turquoise if they have been previously reported[3]. In the Q-Q plots, the black dots represent observed P-values and the grey line represents expected P-values under the null distribution. The green dots represent observed P-values after excluding the previously identified signals[3]. All P-values are two sided and not adjusted for multiple comparisons.

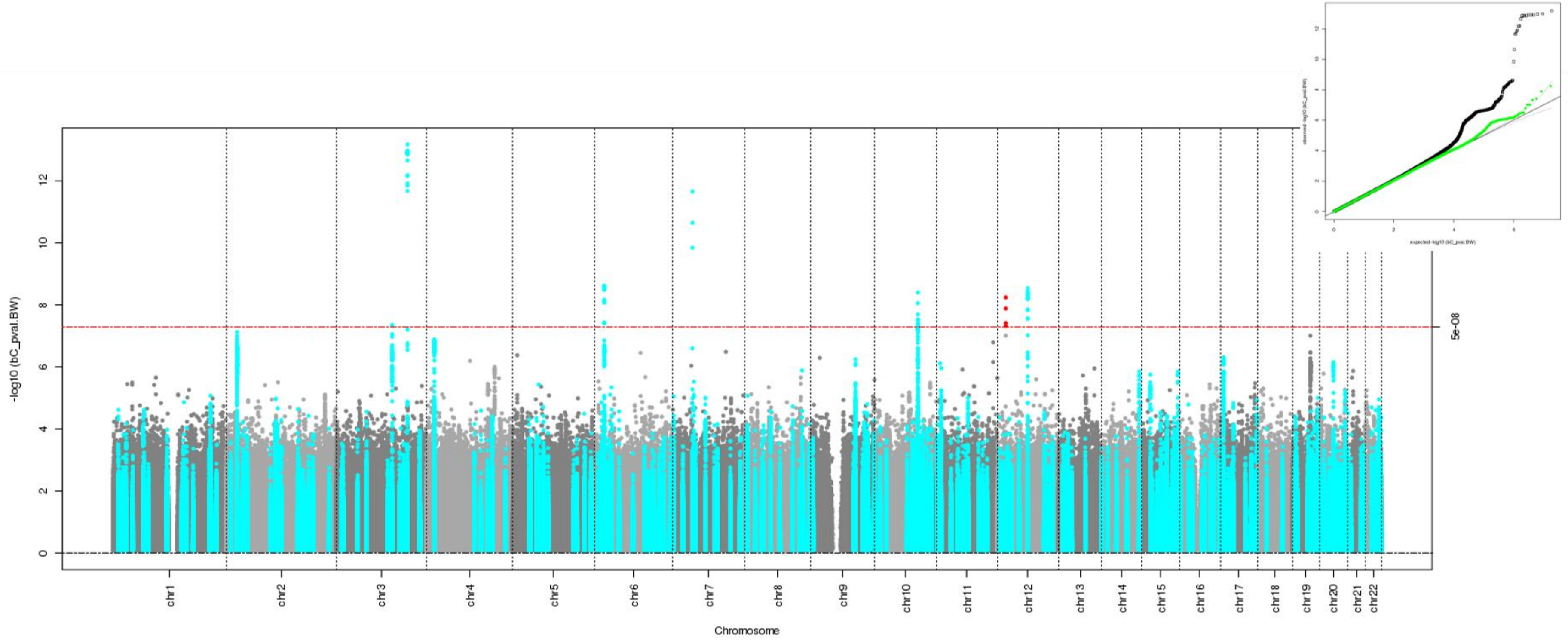

Supplementary Figure 5: Manhattan plot and quantile-quantile (Q-Q) plot for the offspring specific effect estimated using Genomic SEM[6] and no sample overlap between the GWAS of own and offspring birth weight. Point estimates for maternal and offspring effects and their standard errors were estimated using diagonally weighted least squares as implemented in Genomic SEM, and two sided P-values obtained from Z tests on these estimates. The two-sided association P-value, on the –log10 scale, obtained from Genomic SEM for each of the SNPs (y-axis) was plotted against the genomic position (NCBI Build 37; x-axis). Association signals that reached genome-wide significance (P < $5x10^{-8}$) are shown in red if they are novel and turquoise if they have been previously reported[3]. In the Q-Q plots, the black dots represent observed P-values and the grey line represents expected P-values under the null distribution. The green dots represent observed P-values after excluding the previously identified signals[3]. All P-values are two sided and not adjusted for multiple comparisons.

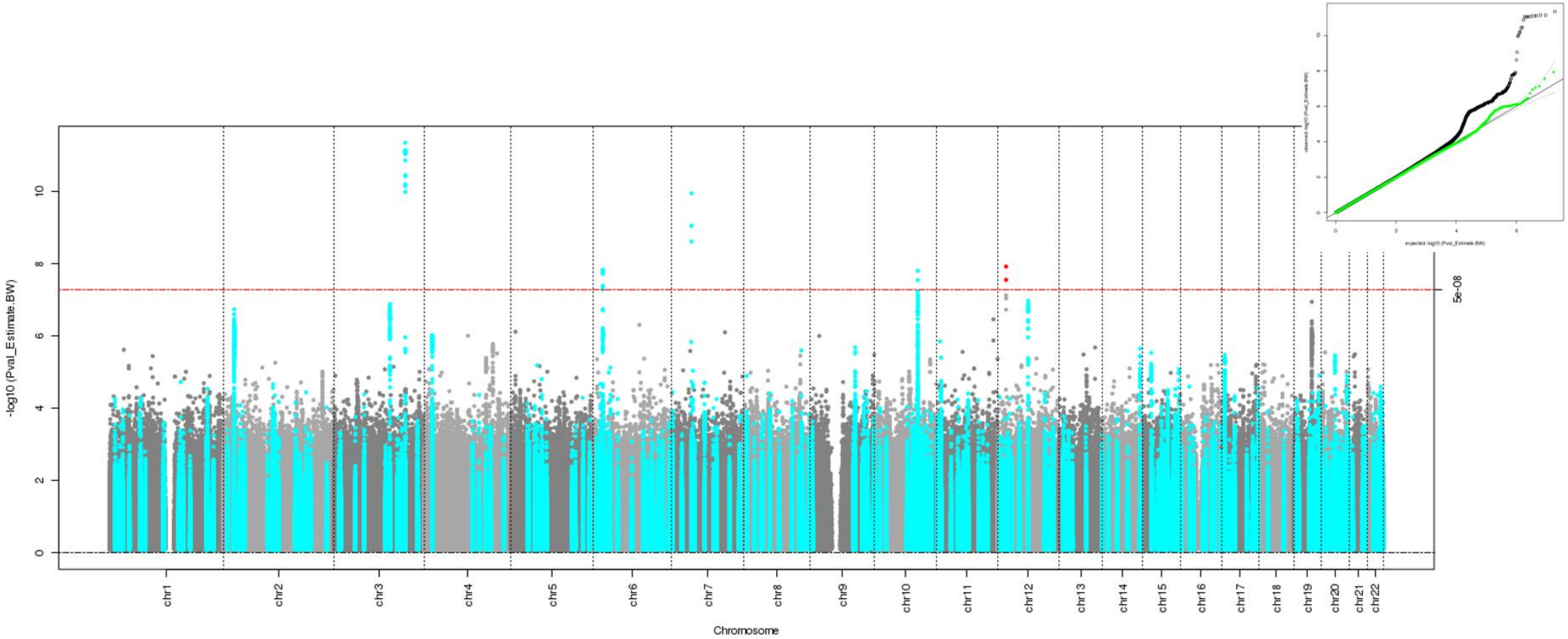

Supplementary Figure 6: Manhattan plot and quantile-quantile (Q-Q) plot for the offspring specific effect estimated using the structural equation model (SEM) with summary statistics and sample overlap between the GWAS of own and offspring birth weight. We calculated a Wald P-value for the offspring genetic effects from the SEM using their effect size estimates and standard errors. The two-sided association P-value, on the –log10 scale, obtained from the SEM for each of the SNPs (y-axis) was plotted against the genomic position (NCBI Build 37; x-axis). Association signals that reached genome-wide significance ($P < 5x10^{-8}$) are shown in red if they are novel and turquoise if they have been previously reported[3]. In the Q-Q plots, the black dots represent observed P-values and the grey line represents expected P-values under the null distribution. The green dots represent observed P-values after excluding the previously identified signals[3]. All P-values are two sided and not adjusted for multiple comparisons.

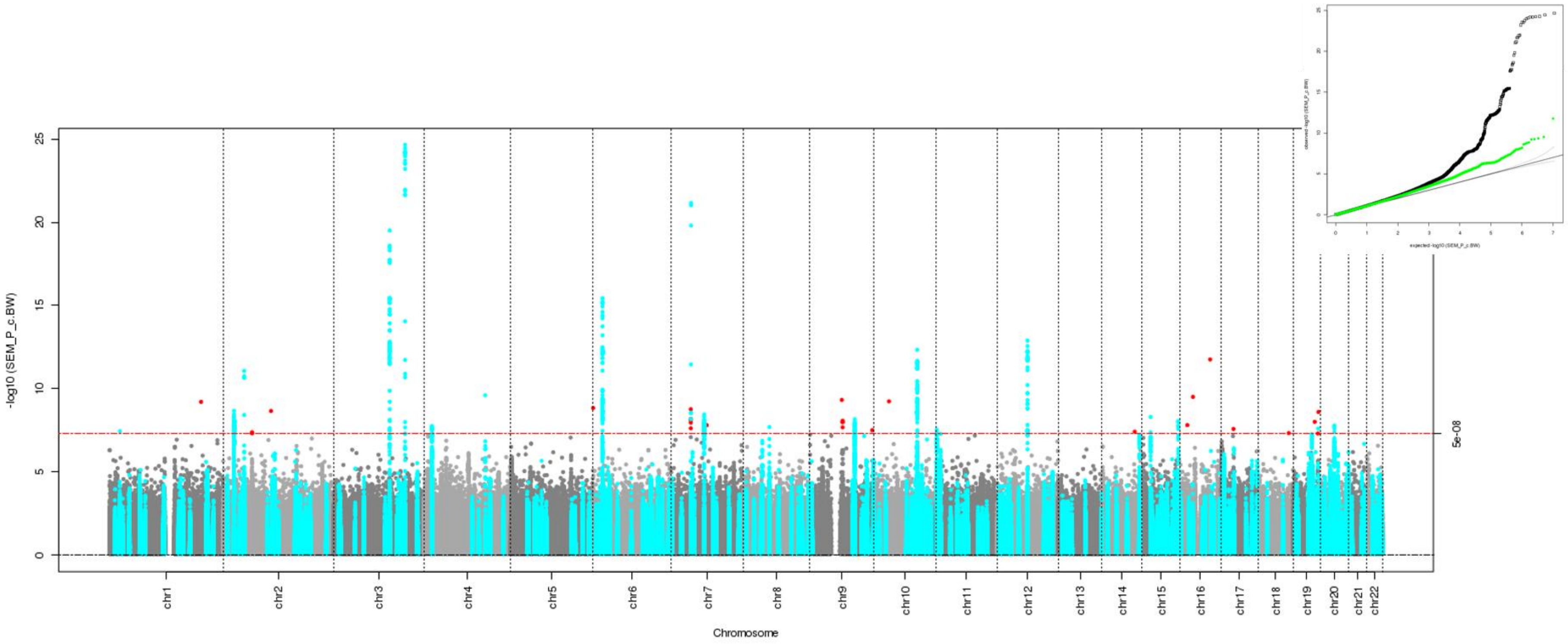

Supplementary Figure 7: Manhattan plot and quantile-quantile (Q-Q) plot for the offspring specific effect estimated using the linear approximation of the structural equation model (SEM) and sample overlap between the GWAS of own and offspring birth weight. We calculated a Z test for the offspring genetic effects using their effect size estimates and standard errors. The two-sided association P-value, on the –log10 scale, obtained from the linear approximation of the SEM for each of the SNPs (y-axis) was plotted against the genomic position (NCBI Build 37; x-axis). Association signals that reached genome-wide significance ($P < 5x10^{-8}$) are shown in red if they are novel and turquoise if they have been previously reported[3]. In the Q-Q plots, the black dots represent observed P-values and the grey line represents expected P-values under the null distribution. The green dots represent observed P-values after excluding the previously identified signals[3]. All P-values are two sided and not adjusted for multiple comparisons.

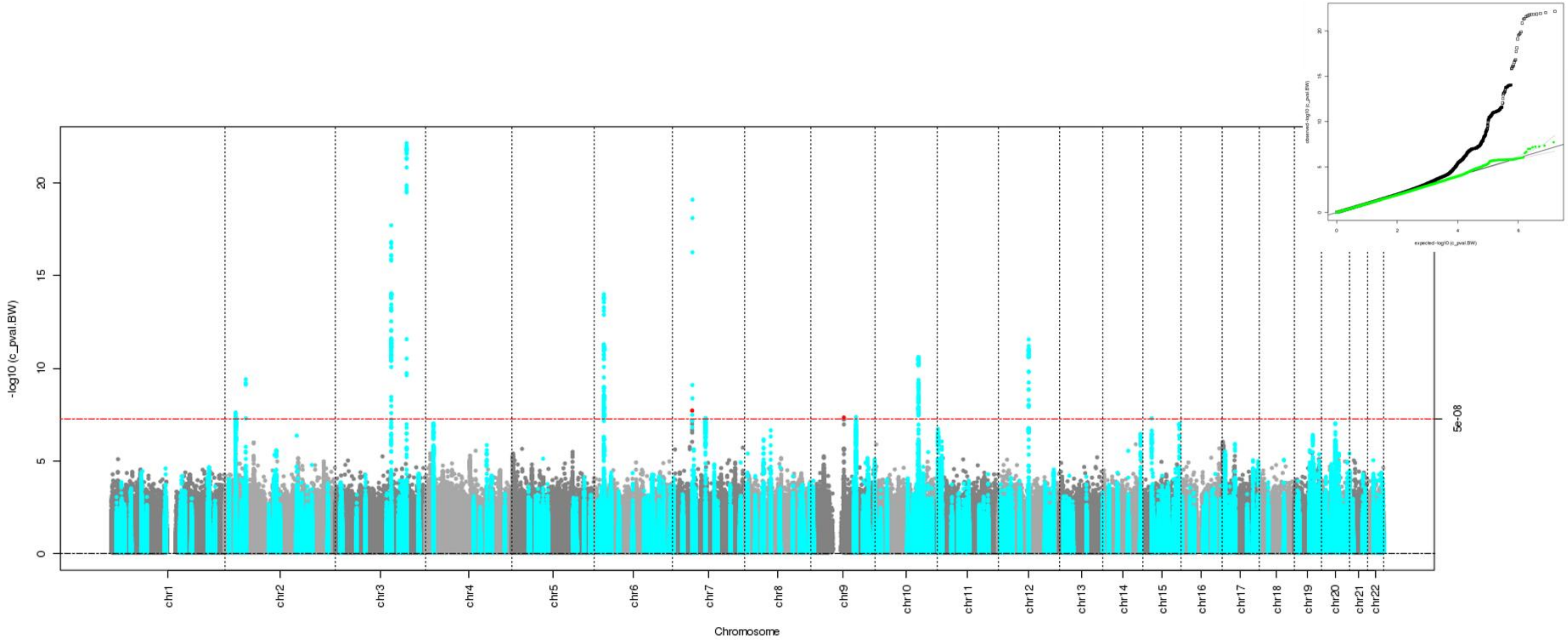

Supplementary Figure 8: Manhattan plot and quantile-quantile (Q-Q) plot for the offspring specific effect estimated using MTAG[4] and sample overlap between the GWAS of own and offspring birth weight. MTAG calculates a Z test using effect size estimates and standard errors. The two-sided association P-value, on the –log10 scale, obtained from MTAG for each of the SNPs (y-axis) was plotted against the genomic position (NCBI Build 37; x-axis). Association signals that reached genome-wide significance ($P < 5x10^{-8}$) are shown in red if they are novel and turquoise if they have been previously reported[3]. In the Q-Q plots, the black dots represent observed P-values and the grey line represents expected P-values under the null distribution. The green dots represent observed P-values after excluding the previously identified signals[3]. All P-values are two sided and not adjusted for multiple comparisons.

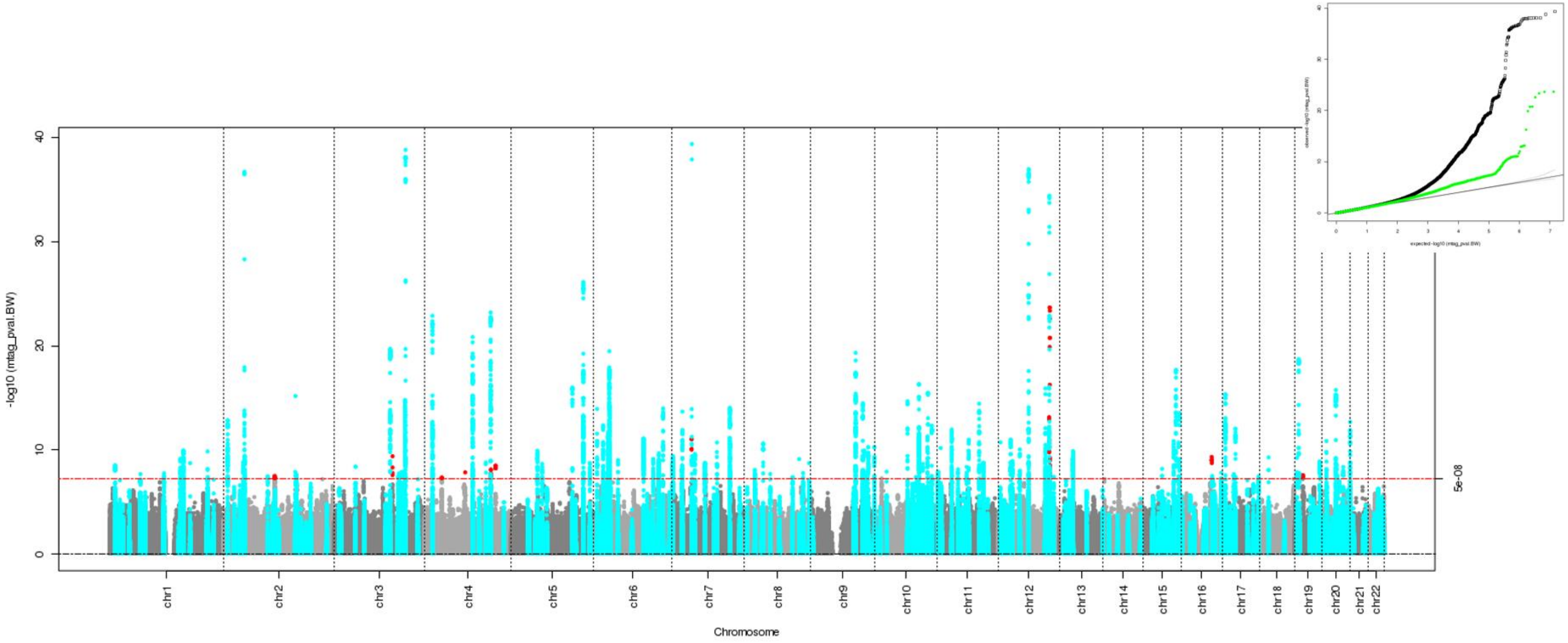

Supplementary Figure 9: Manhattan plot and quantile-quantile (Q-Q) plot for the offspring specific effect estimated using mtCOJO[5] and sample overlap between the GWAS of own and offspring birth weight. mtCOJO calculates a chi-square test using effect size estimates and standard errors. The two-sided association P-value, on the –log10 scale, obtained from mtCOJO for each of the SNPs (y-axis) was plotted against the genomic position (NCBI Build 37; x-axis). Association signals that reached genome-wide significance ($P < 5x10^{-8}$) are shown in red if they are novel and turquoise if they have been previously reported[3]. In the Q-Q plots, the black dots represent observed P-values and the grey line represents expected P-values under the null distribution. The green dots represent observed P-values after excluding the previously identified signals[3]. All P-values are two sided and not adjusted for multiple comparisons.

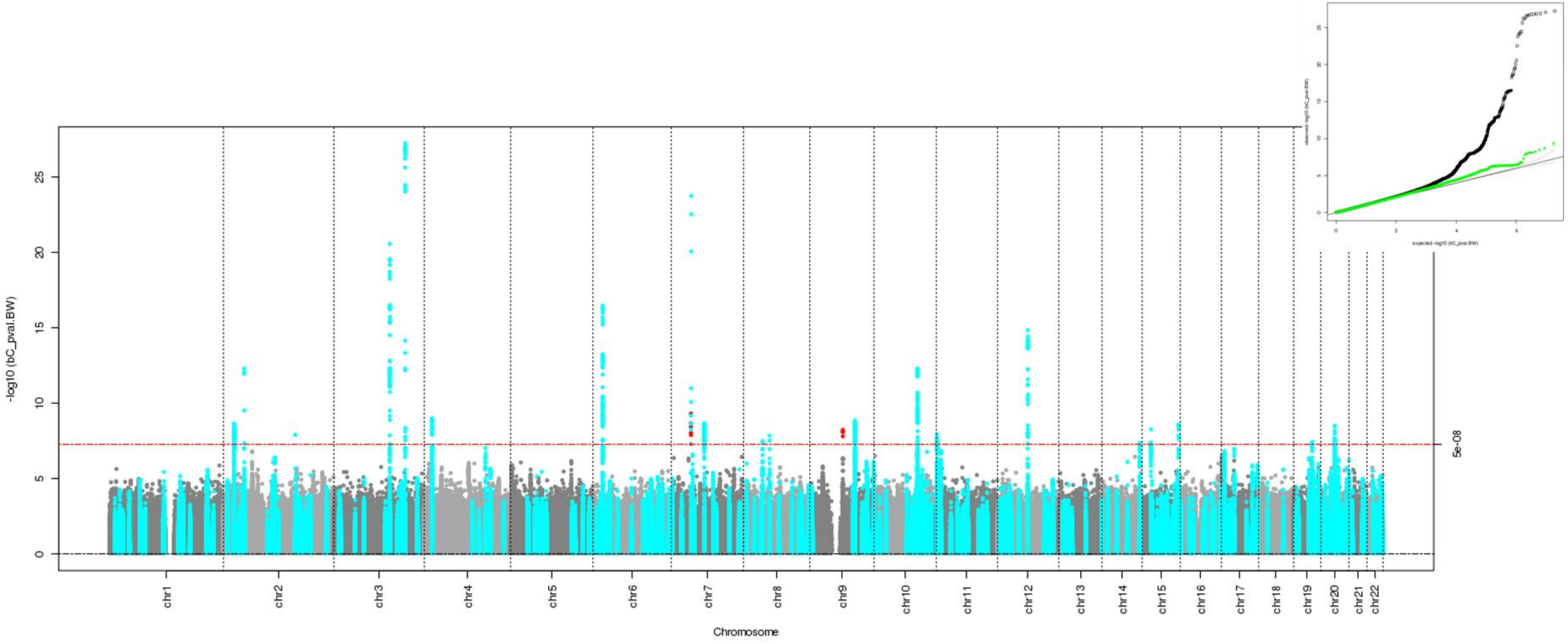

Supplementary Figure 10: Manhattan plot and quantile-quantile (Q-Q) plot for the offspring specific effect estimated Genomic SEM[6] and sample overlap between the GWAS of own and offspring birth weight. Point estimates for maternal and offspring effects and their standard errors were estimated using diagonally weighted least squares as implemented in Genomic SEM, and two sided P-values obtained from Z tests on these estimates. The two-sided association P-value, on the –log10 scale, obtained from Genomic SEM for each of the SNPs (y-axis) was plotted against the genomic position (NCBI Build 37; x-axis). Association signals that reached genome-wide significance ($P < 5x10^{-8}$) are shown in red if they are novel and turquoise if they have been previously reported[3]. In the Q-Q plots, the black dots represent observed P-values and the grey line represents expected P-values under the null distribution. The green dots represent observed P-values after excluding the previously identified signals[3]. All P-values are two sided and not adjusted for multiple comparisons.

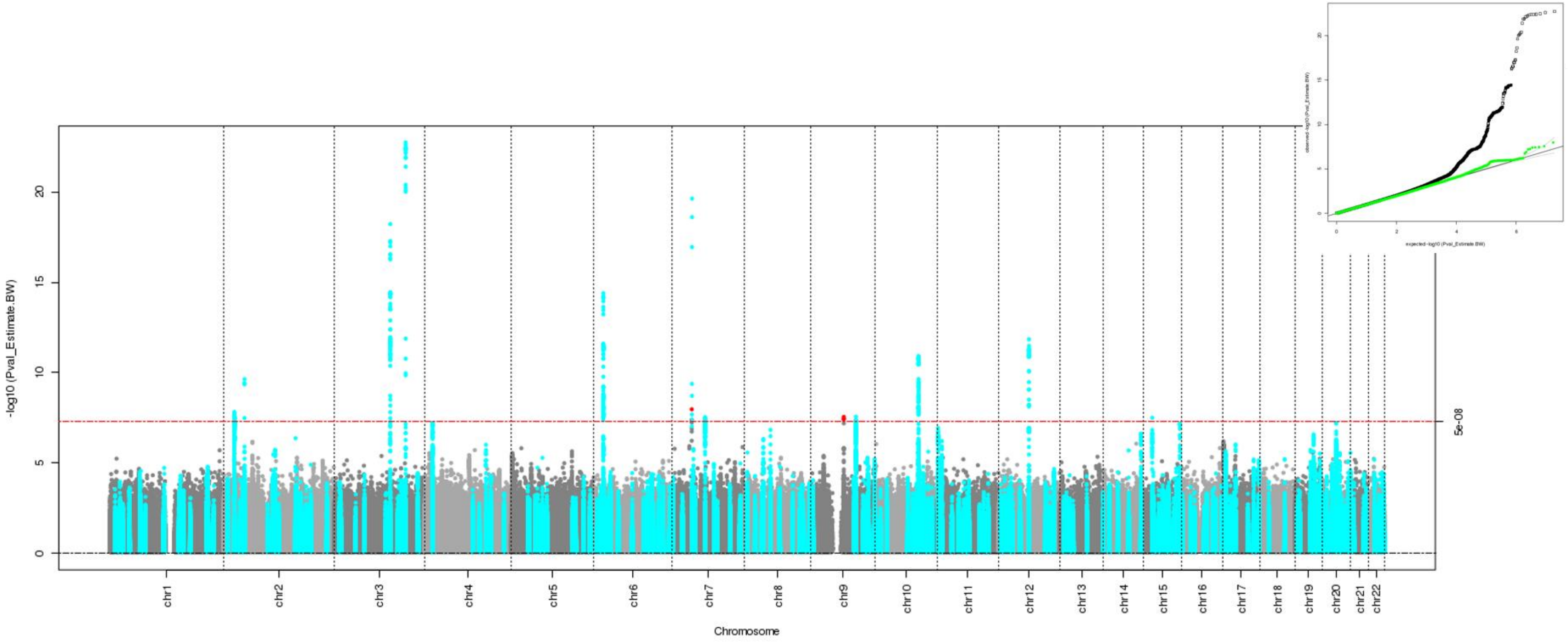

Supplementary Figure 11: Manhattan plot and quantile-quantile (Q-Q) plot for the maternal specific effect estimated using the structural equation model (SEM) with summary statistics and no sample overlap between the GWAS of own and offspring birth weight. We calculated a Wald P-value for the maternal genetic effects from the SEM using their effect size estimates and standard errors. The two-sided association P-value, on the –log10 scale, obtained from the SEM for each of the SNPs (y-axis) was plotted against the genomic position (NCBI Build 37; x-axis). Association signals that reached genome-wide significance ($P < 5x10^{-8}$) are shown in red if they are novel and turquoise if they have been previously reported[3]. In the Q-Q plots, the black dots represent observed P-values and the grey line represents expected P-values under the null distribution. The green dots represent observed P-values after excluding the previously identified signals[3]. All P-values are two sided and not adjusted for multiple comparisons.

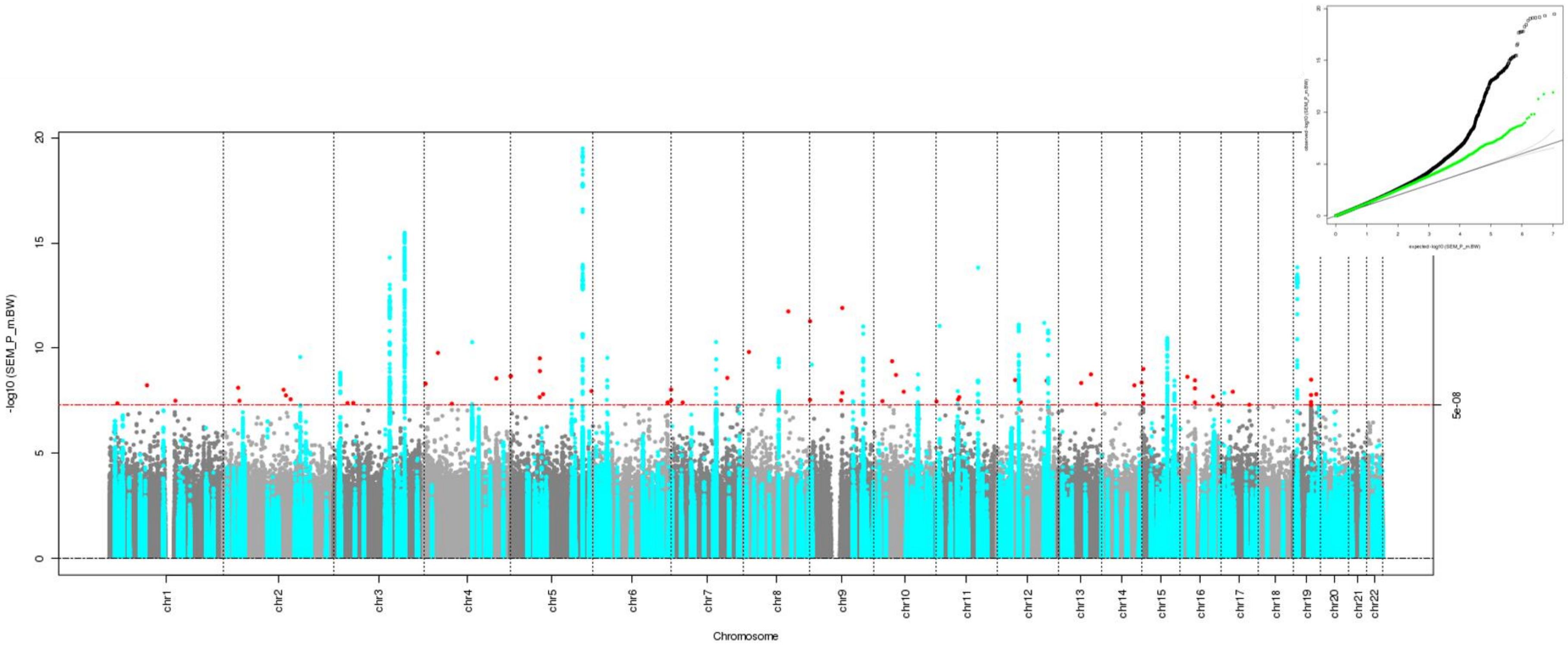

Supplementary Figure 12: Manhattan plot and quantile-quantile (Q-Q) plot for the maternal specific effect estimated using the linear approximation of the structural equation model (SEM) and no sample overlap between the GWAS of own and offspring birth weight. We calculated a Z test for the maternal genetic effects using their effect size estimates and standard errors. The two-sided association P-value, on the –log10 scale, obtained from the linear approximation of the SEM for each of the SNPs (y-axis) was plotted against the genomic position (NCBI Build 37; x-axis). Association signals that reached genome-wide significance ($P < 5x10^{-8}$) are shown in red if they are novel and turquoise if they have been previously reported[3]. In the Q-Q plots, the black dots represent observed P-values and the grey line represents expected P-values under the null distribution. The green dots represent observed P-values after excluding the previously identified signals[3]. All P-values are two sided and not adjusted for multiple comparisons.

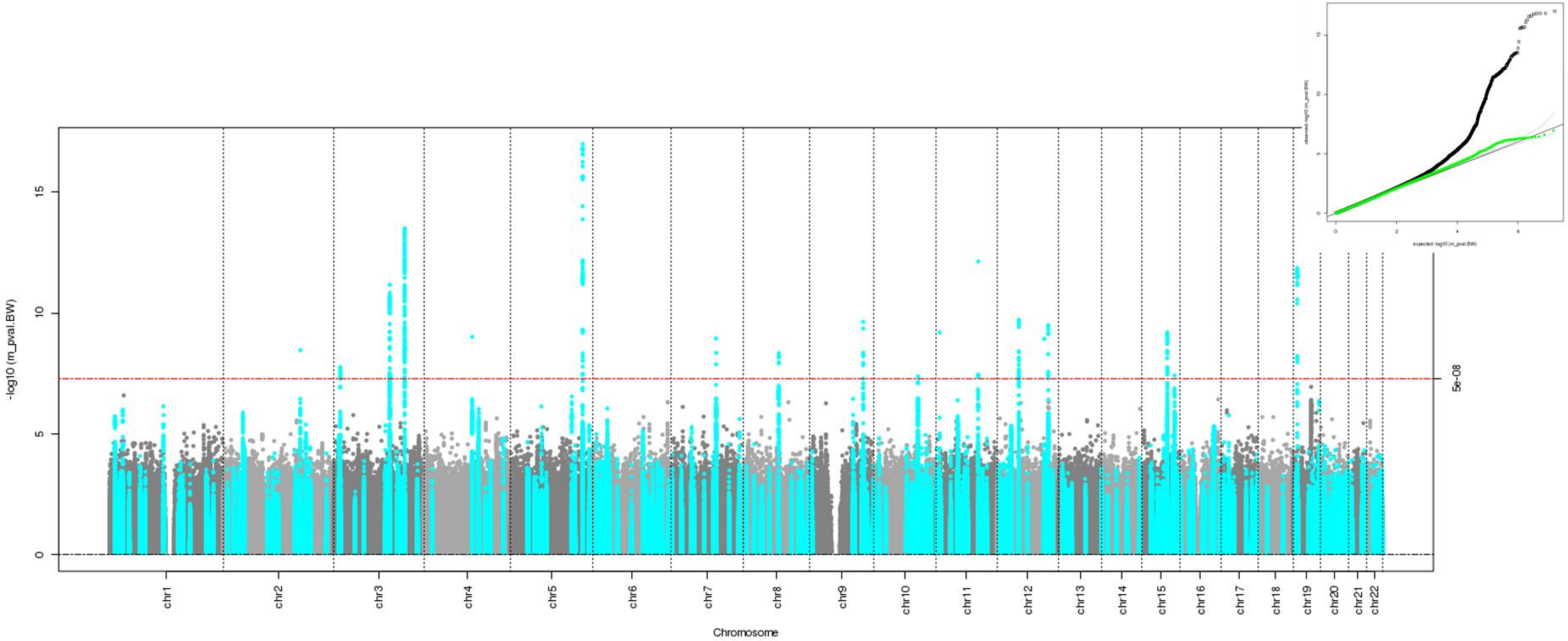

Supplementary Figure 13: Manhattan plot and quantile-quantile (Q-Q) plot for the maternal specific effect estimated using MTAG[4] and no sample overlap between the GWAS of own and offspring birth weight. MTAG calculates a Z test using effect size estimates and standard errors. The two-sided association P-value, on the –log10 scale, obtained from MTAG for each of the SNPs (y-axis) was plotted against the genomic position (NCBI Build 37; x-axis). Association signals that reached genome-wide significance ($P < 5x10^{-8}$) are shown in red if they are novel and turquoise if they have been previously reported[3]. In the Q-Q plots, the black dots represent observed P-values and the grey line represents expected P-values under the null distribution. The green dots represent observed P-values after excluding the previously identified signals[3]. All P-values are two sided and not adjusted for multiple comparisons.

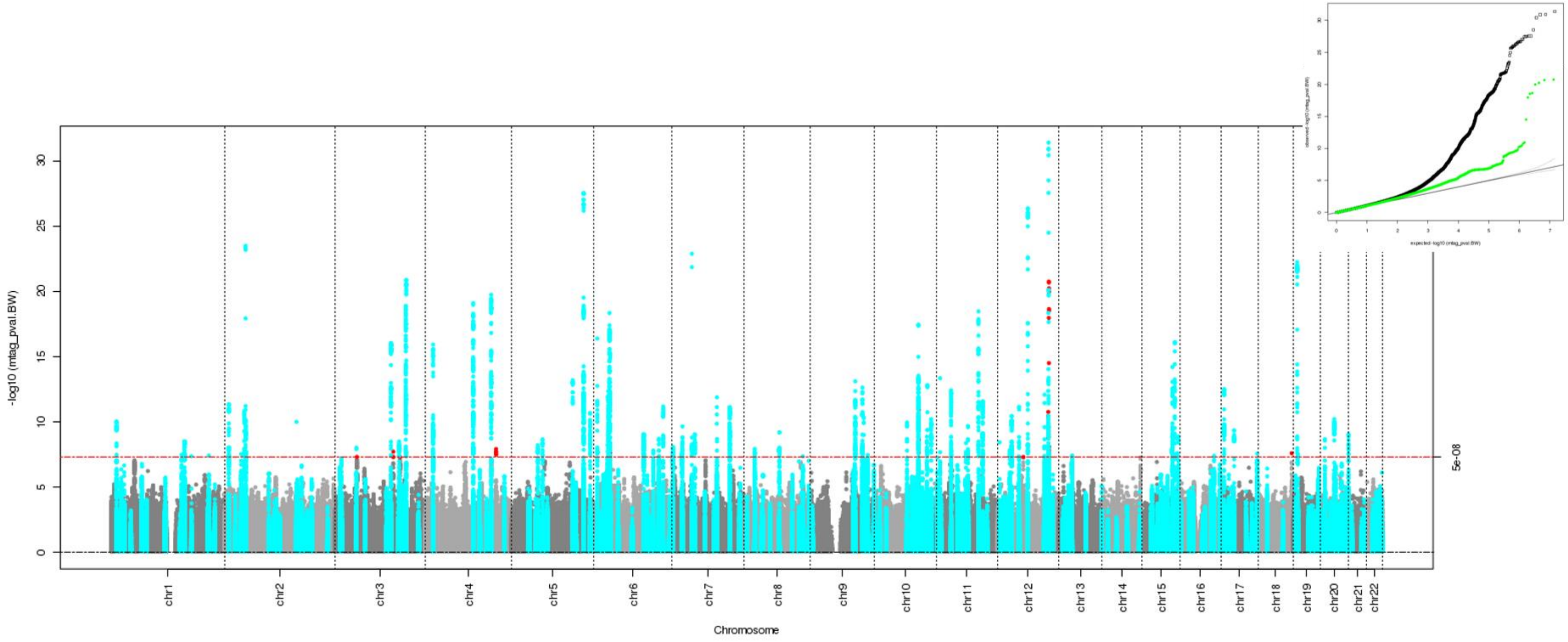

Supplementary Figure 14: Manhattan plot and quantile-quantile (Q-Q) plot for the maternal specific effect estimated using mtCOJO[5] and no sample overlap between the GWAS of own and offspring birth weight. mtCOJO calculates a chi-square test using effect size estimates and standard errors. The two-sided association P-value, on the –log10 scale, obtained from mtCOJO for each of the SNPs (y-axis) was plotted against the genomic position (NCBI Build 37; x-axis). Association signals that reached genome-wide significance ($P < 5\text{x}10^{-8}$) are shown in red if they are novel and turquoise if they have been previously reported[3]. In the Q-Q plots, the black dots represent observed P-values and the grey line represents expected P-values under the null distribution. The green dots represent observed P-values after excluding the previously identified signals[3]. All P-values are two sided and not adjusted for multiple comparisons.

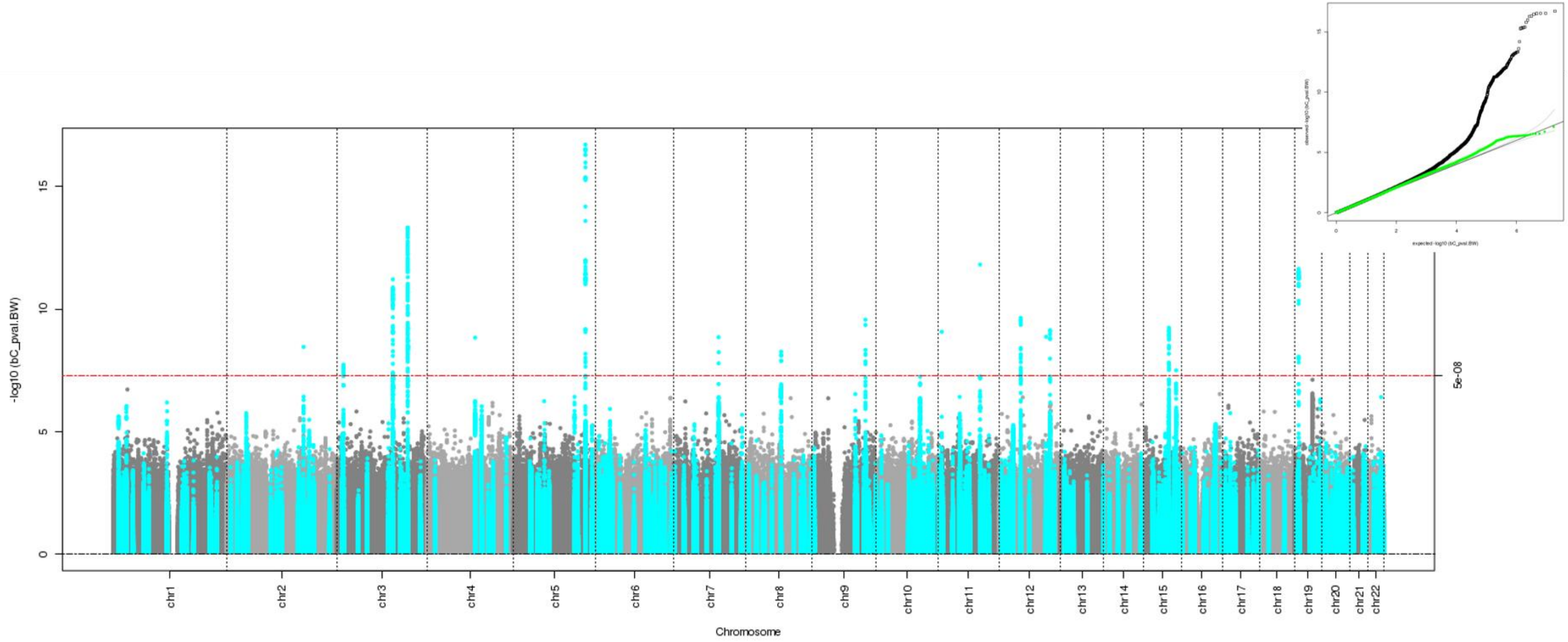

Supplementary Figure 15: Manhattan plot and quantile-quantile (Q-Q) plot for the maternal specific effect estimated using Genomic SEM[6] and no sample overlap between the GWAS of own and offspring birth weight. Point estimates for maternal and offspring effects and their standard errors were estimated using diagonally weighted least squares as implemented in Genomic SEM, and two sided P-values obtained from Z tests on these estimates. The two-sided association P-value, on the –log10 scale, obtained from Genomic SEM for each of the SNPs (y-axis) was plotted against the genomic position (NCBI Build 37; x-axis). Association signals that reached genome-wide significance ($P < 5x10^{-8}$) are shown in red if they are novel and turquoise if they have been previously reported[3]. In the Q-Q plots, the black dots represent observed P-values and the grey line represents expected P-values under the null distribution. The green dots represent observed P-values after excluding the previously identified signals[3]. All P-values are two sided and not adjusted for multiple comparisons.

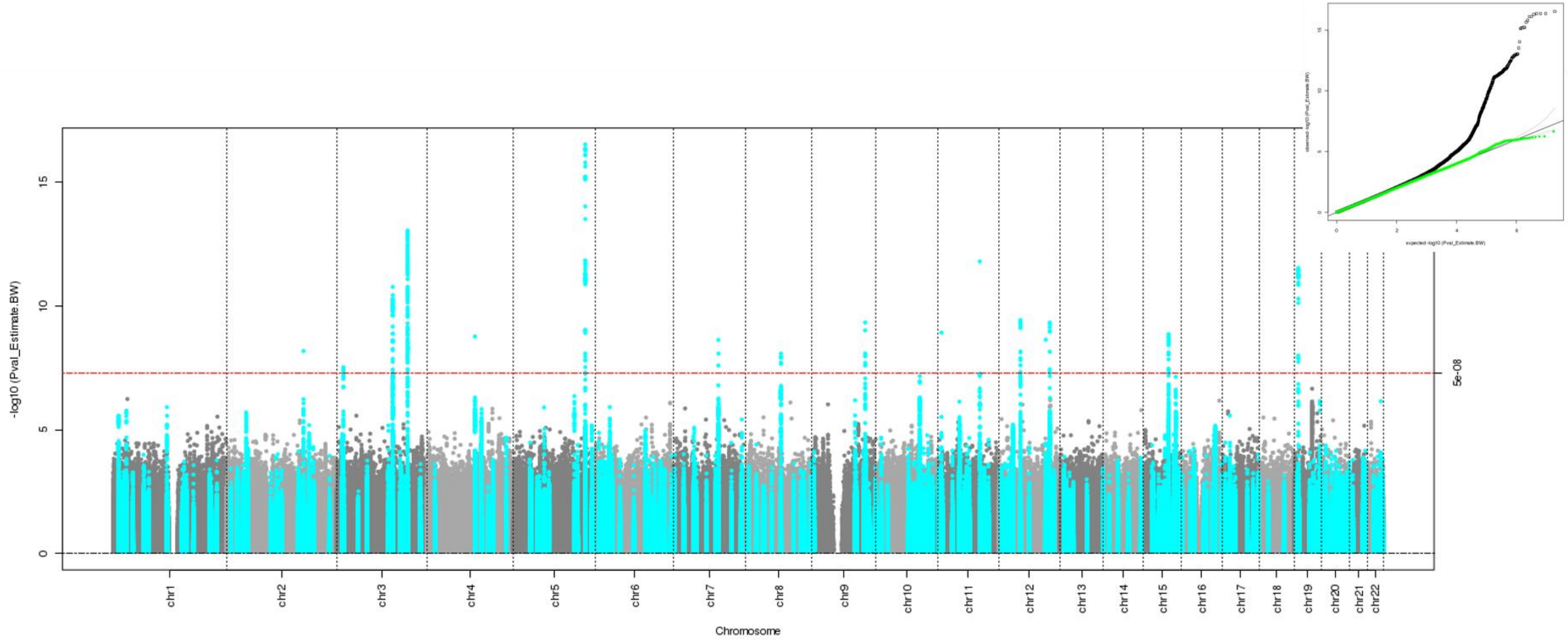

Supplementary Figure 16: Manhattan plot and quantile-quantile (Q-Q) plot for the maternal specific effect estimated using the structural equation model (SEM) with summary statistics and sample overlap between the GWAS of own and offspring birth weight. We calculated a Wald P-value for the maternal genetic effects from the SEM using their effect size estimates and standard errors. The two-sided association P-value, on the –log10 scale, obtained from the SEM for each of the SNPs (y-axis) was plotted against the genomic position (NCBI Build 37; x-axis). Association signals that reached genome-wide significance ($P < 5x10^{-8}$) are shown in red if they are novel and turquoise if they have been previously reported[3]. In the Q-Q plots, the black dots represent observed P-values and the grey line represents expected P-values under the null distribution. The green dots represent observed P-values after excluding the previously identified signals[3]. All P-values are two sided and not adjusted for multiple comparisons.

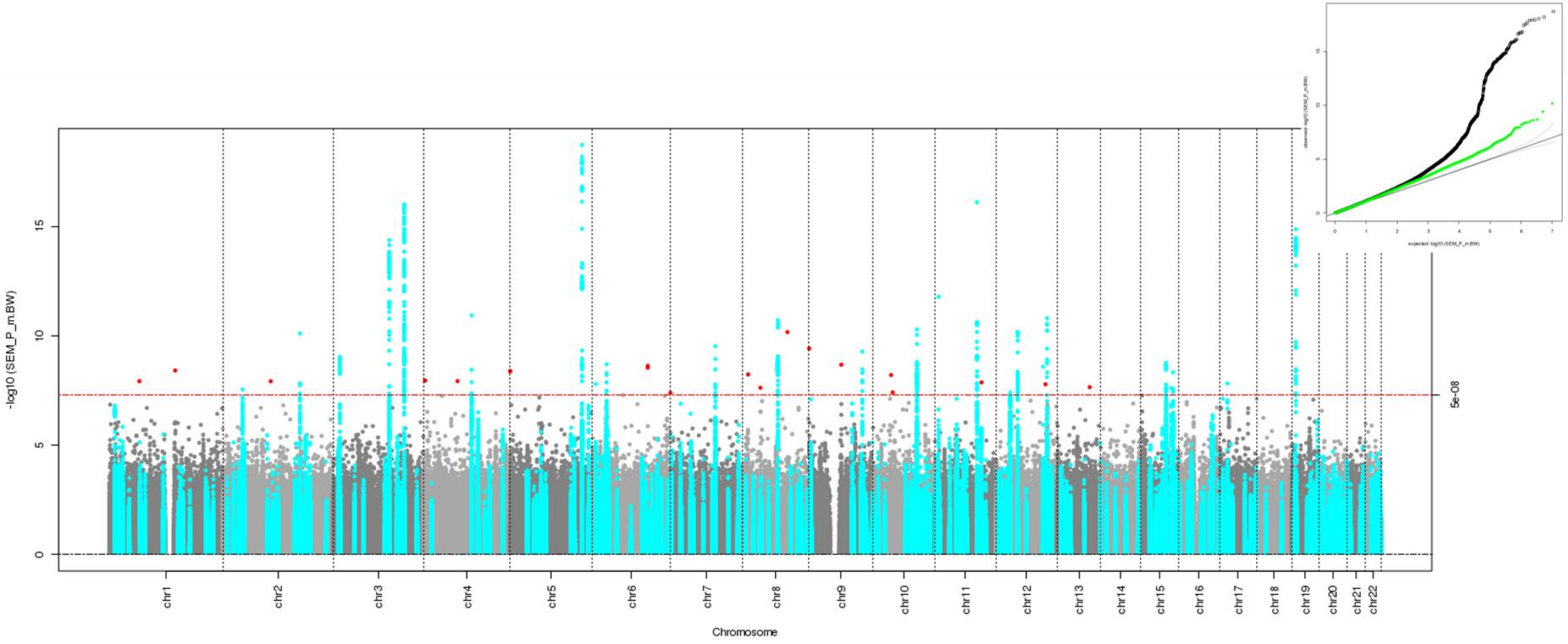

Supplementary Figure 17: Manhattan plot and quantile-quantile (Q-Q) plot for the maternal specific effect estimated using the linear approximation of the structural equation model (SEM) and sample overlap between the GWAS of own and offspring birth weight. We calculated a Z test for the maternal genetic effects using their effect size estimates and standard errors. The two-sided association P-value, on the –log10 scale, obtained from the linear approximation of the SEM for each of the SNPs (y-axis) was plotted against the genomic position (NCBI Build 37; x-axis). Association signals that reached genome-wide significance ($P < 5x10^{-8}$) are shown in red if they are novel and turquoise if they have been previously reported[3]. In the Q-Q plots, the black dots represent observed P-values and the grey line represents expected P-values under the null distribution. The green dots represent observed P-values after excluding the previously identified signals[3]. All P-values are two sided and not adjusted for multiple comparisons.

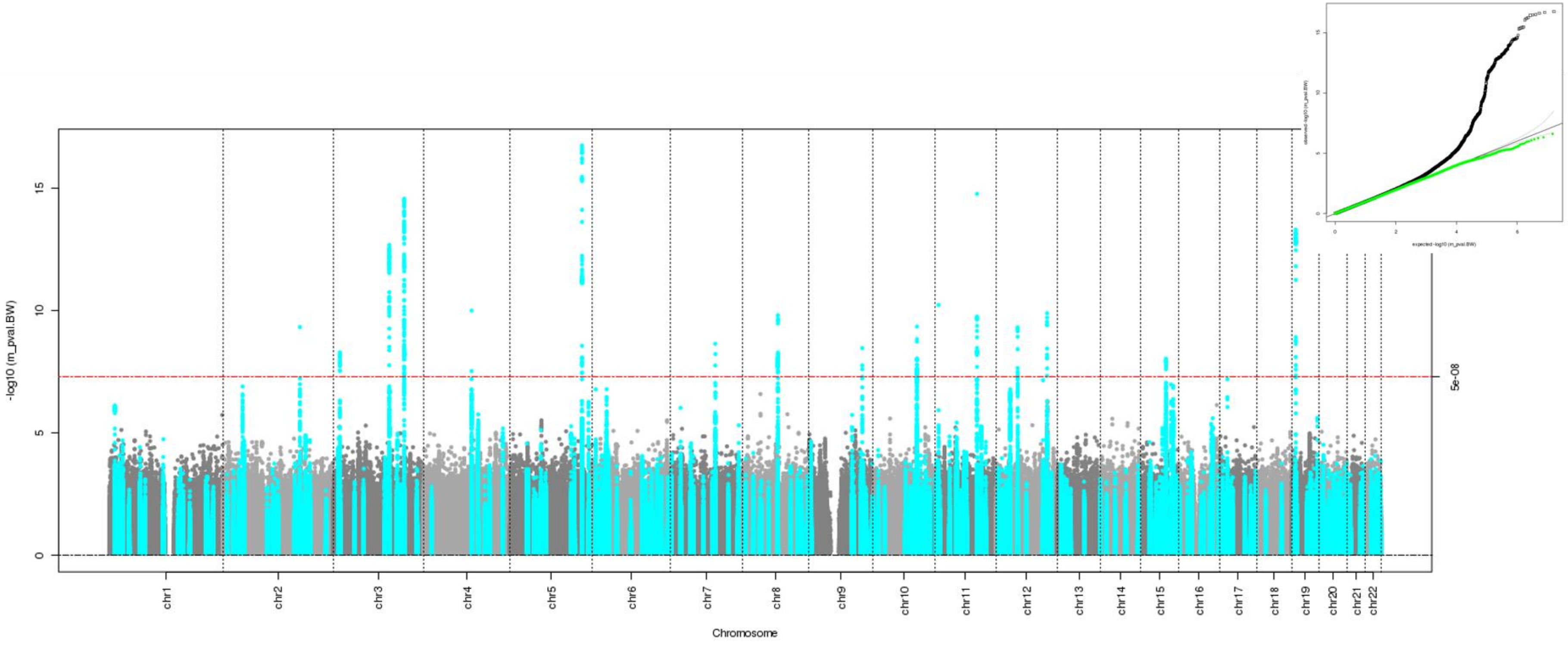

Supplementary Figure 18: Manhattan plot and quantile-quantile (Q-Q) plot for the maternal specific effect estimated using MTAG[4] and sample overlap between the GWAS of own and offspring birth weight. MTAG calculates a Z test using effect size estimates and standard errors. The two-sided association P-value, on the –log10 scale, obtained from MTAG for each of the SNPs (y-axis) was plotted against the genomic position (NCBI Build 37; x-axis). Association signals that reached genome-wide significance ($P < 5x10^{-8}$) are shown in red if they are novel and turquoise if they have been previously reported[3]. In the Q-Q plots, the black dots represent observed P-values and the grey line represents expected P-values under the null distribution. The green dots represent observed P-values after excluding the previously identified signals[3]. All P-values are two sided and not adjusted for multiple comparisons.

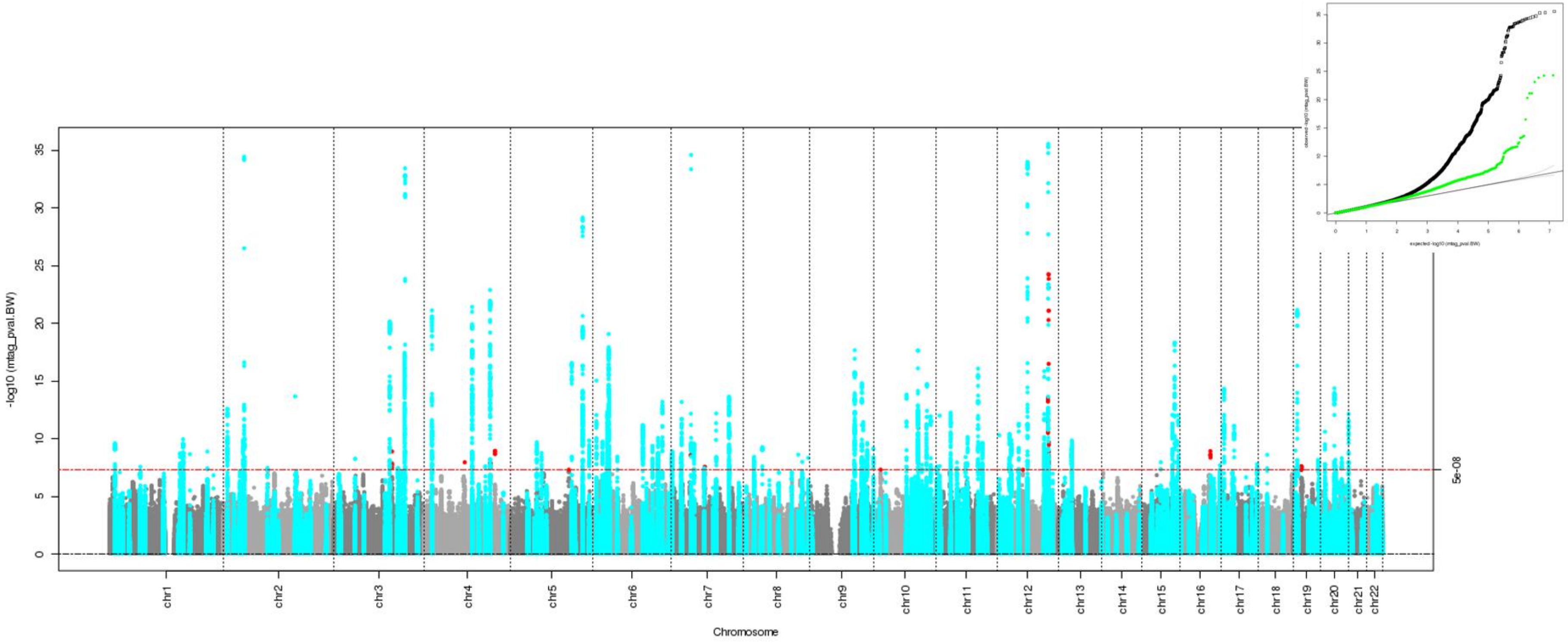

Supplementary Figure 19: Manhattan plot and quantile-quantile (Q-Q) plot for the maternal specific effect estimated mtCOJO[5] and sample overlap between the GWAS of own and offspring birth weight. mtCOJO calculates a chi-square test using effect size estimates and standard errors. The two-sided association P-value, on the –log10 scale, obtained from mtCOJO for each of the SNPs (y-axis) was plotted against the genomic position (NCBI Build 37; x-axis). Association signals that reached genome-wide significance ($P < 5x10^{-8}$) are shown in red if they are novel and turquoise if they have been previously reported[3]. In the Q-Q plots, the black dots represent observed P-values and the grey line represents expected P-values under the null distribution. The green dots represent observed P-values after excluding the previously identified signals[3]. All P-values are two sided and not adjusted for multiple comparisons.

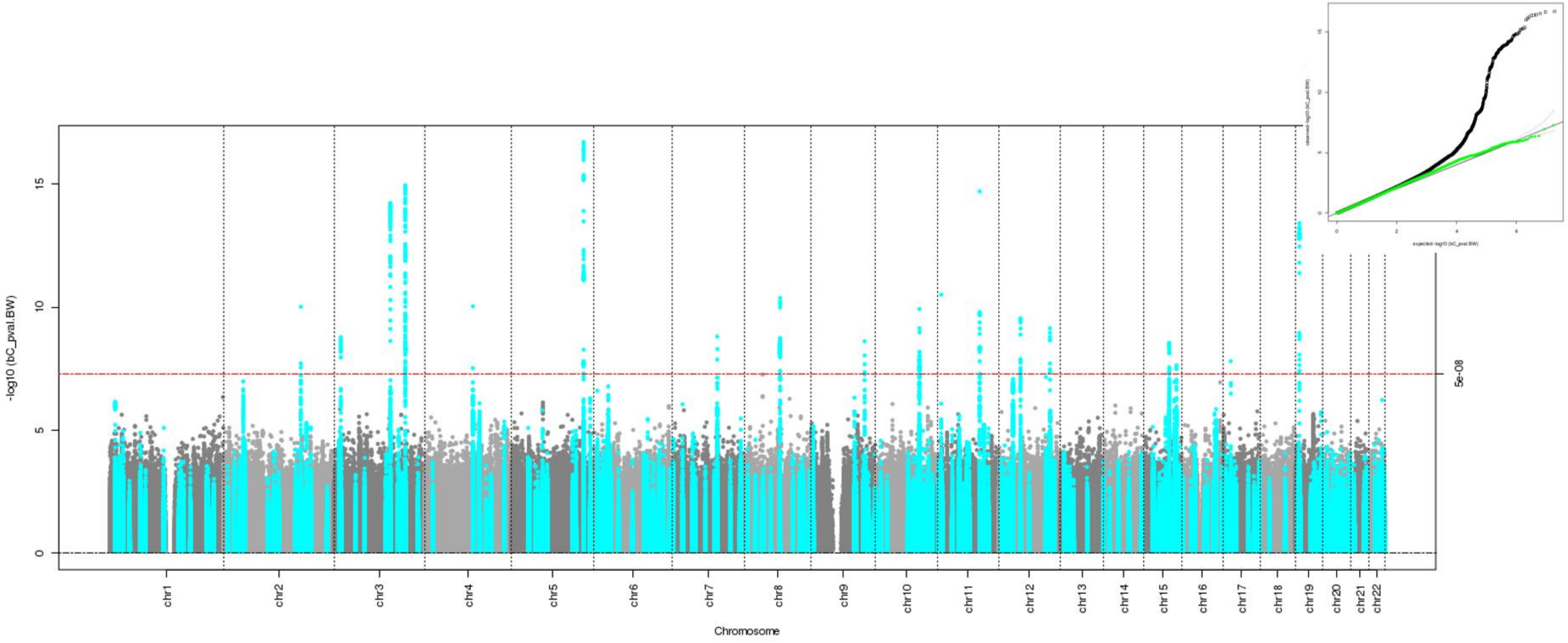

Supplementary Figure 20: Manhattan plot and quantile-quantile (Q-Q) plot for the maternal specific effect estimated using Genomic SEM[6] and sample overlap between the GWAS of own and offspring birth weight. Point estimates for maternal and offspring effects and their standard errors were estimated using diagonally weighted least squares as implemented in Genomic SEM, and two sided P-values obtained from Z tests on these estimates. The two-sided association P-value, on the –log10 scale, obtained from Genomic SEM for each of the SNPs (y-axis) was plotted against the genomic position (NCBI Build 37; x-axis). Association signals that reached genome-wide significance ($P < 5x10^{-8}$) are shown in red if they are novel and turquoise if they have been previously reported[3]. In the Q-Q plots, the black dots represent observed P-values and the grey line represents expected P-values under the null distribution. The green dots represent observed P-values after excluding the previously identified signals[3]. All P-values are two sided and not adjusted for multiple comparisons.

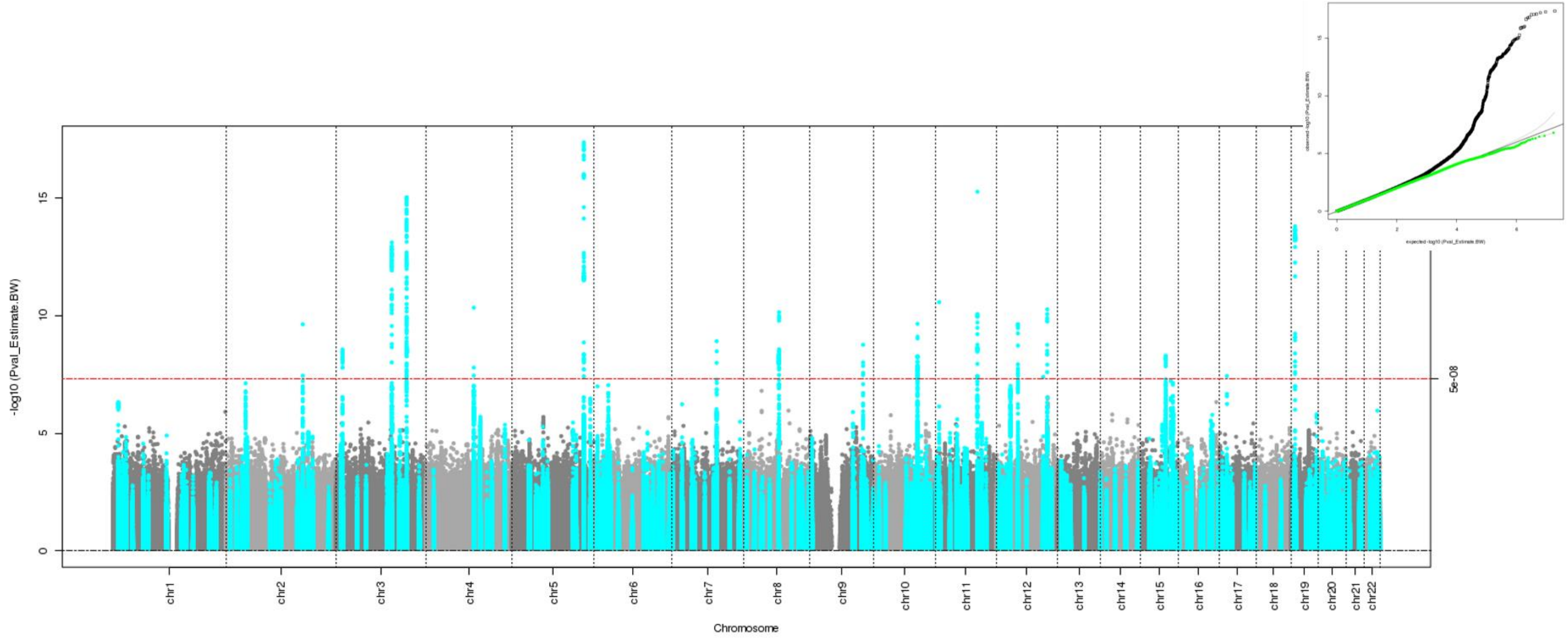

Supplementary Figure 21: Manhattan plot and quantile-quantile (Q-Q) plot for the fertility GWAS estimating the genetic effects on number of children mothered and fathered and the number of siblings estimated using BOLT-LMM. The two-sided association P-value, on the –log10 scale, obtained from BOLT-LMM for each of the SNPs (y-axis) was plotted against the genomic position (NCBI Build 37; x-axis). Association signals that reached genome-wide significance ($P < 5x10^{-8}$) are shown in red. In the Q-Q plots, the black dots represent observed P-values and the grey line represents expected P-values under the null distribution. All P-values are two sided and not adjusted for multiple comparisons.

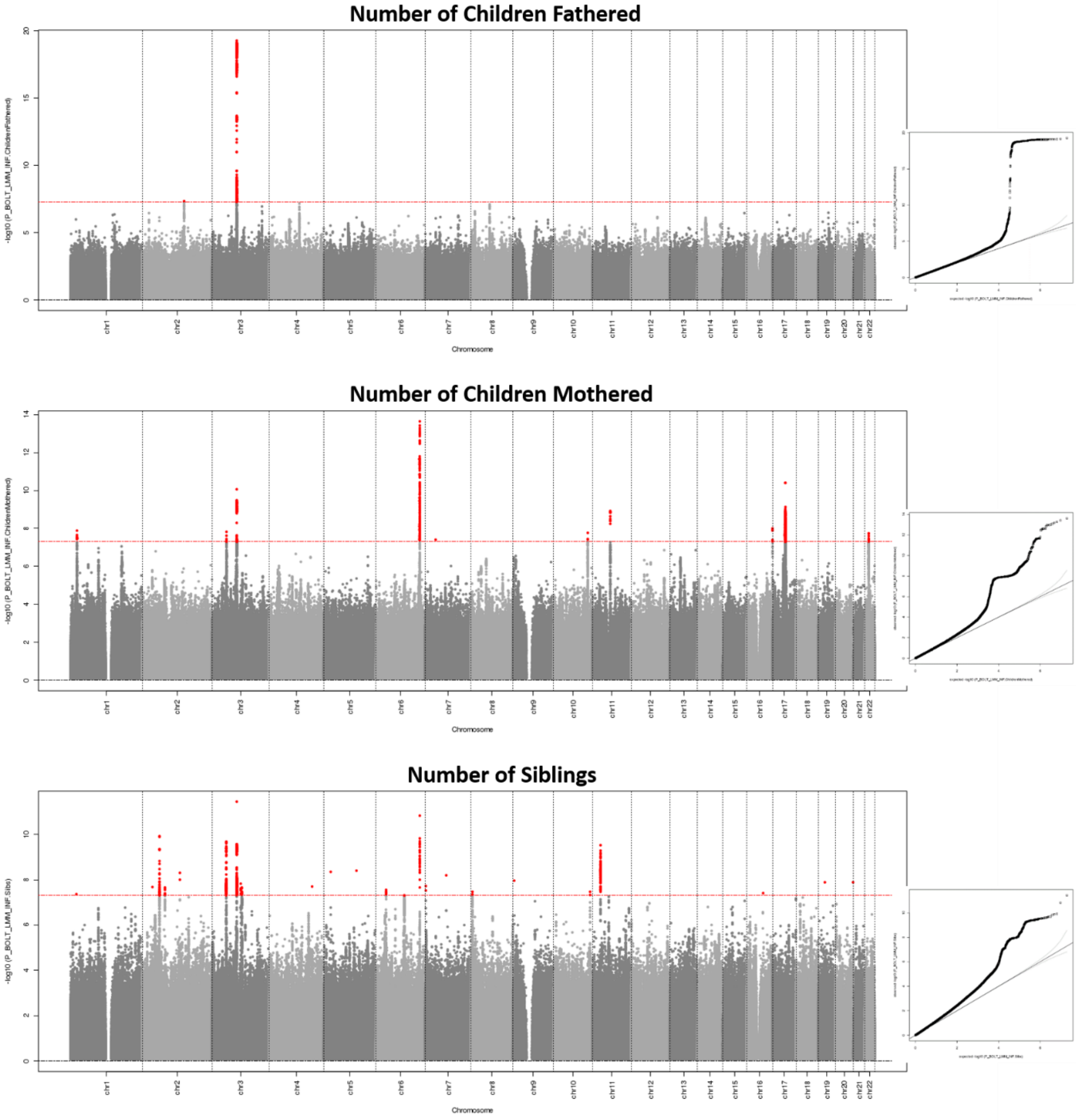

Supplementary Figure 22: Manhattan plot and quantile-quantile (Q-Q) plot for the fertility GWAS estimating maternal and offspring specific genetic effects using Genomic SEM. Point estimates for maternal and offspring effects and their standard errors were estimated using diagonally weighted least squares as implemented in Genomic SEM, and two sided P-values obtained from Z tests on these estimates. The two-sided association P-value, on the –log10 scale, obtained from Genomic SEM for each of the SNPs (y-axis) was plotted against the genomic position (NCBI Build 37; x-axis). Association signals that reached genome-wide significance ($P < 5x10^{-8}$) are shown in red. In the Q-Q plots, the black dots represent observed P-values and the grey line represents expected P-values under the null distribution. All P-values are two sided and not adjusted for multiple comparisons.

Female specific effects

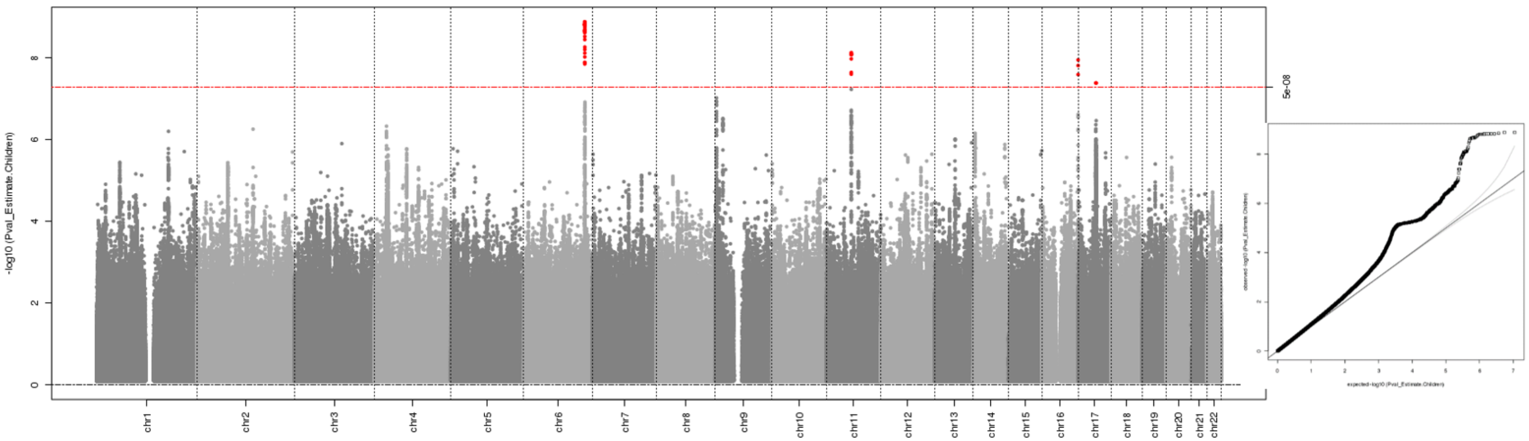

Sibling specific effects

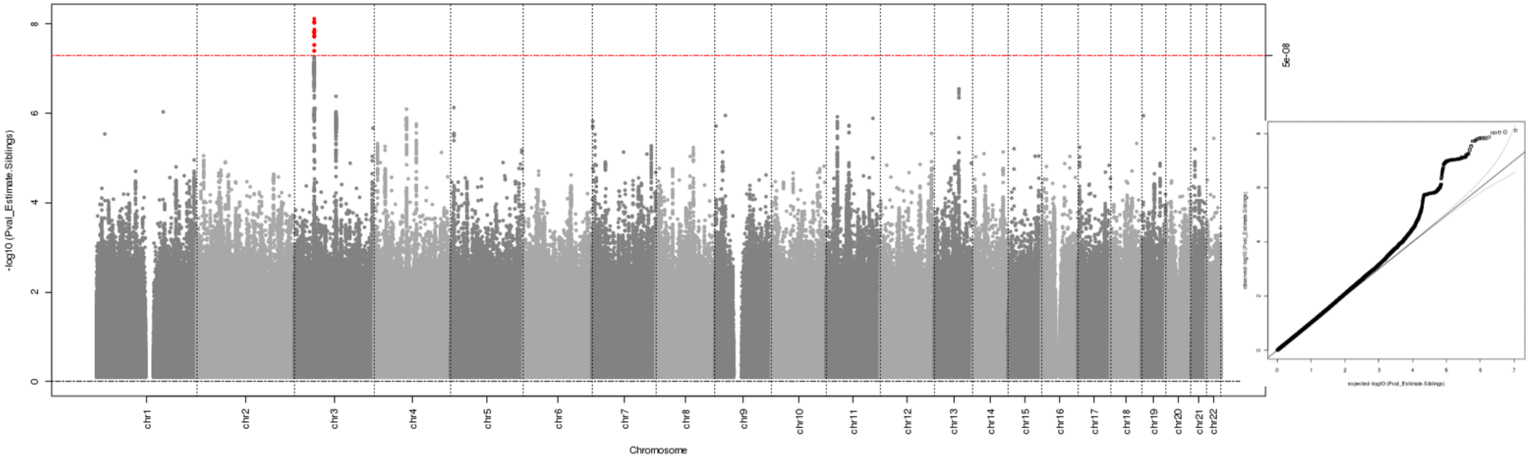

Supplementary Figure 23: Genetic correlation between male and female fertility and sibling specific effects and traits related to development, reproduction, behaviour, neuropsychiatric disorders and anthropometry. Genetic correlations (Rg) were calculated using LD score regression[7], conducted in LD Hub[8]. The traits were chosen based on those used in Barban et al[9]. The point indicates the genetic correlation and the bars indicate the 95% confidence interval; the size of the point is proportional to 1/(standard error)^2. Asterisks indicate that the estimate of genetic correlation is statistically significant after controlling for multiple testing ($P<0.05/22=0.002$). Analyses based on N = 237,768 women reporting how many children they mothered, N = 199,570 men reporting how many children they had fathered and N = 430,466 individuals reporting how many siblings they have.

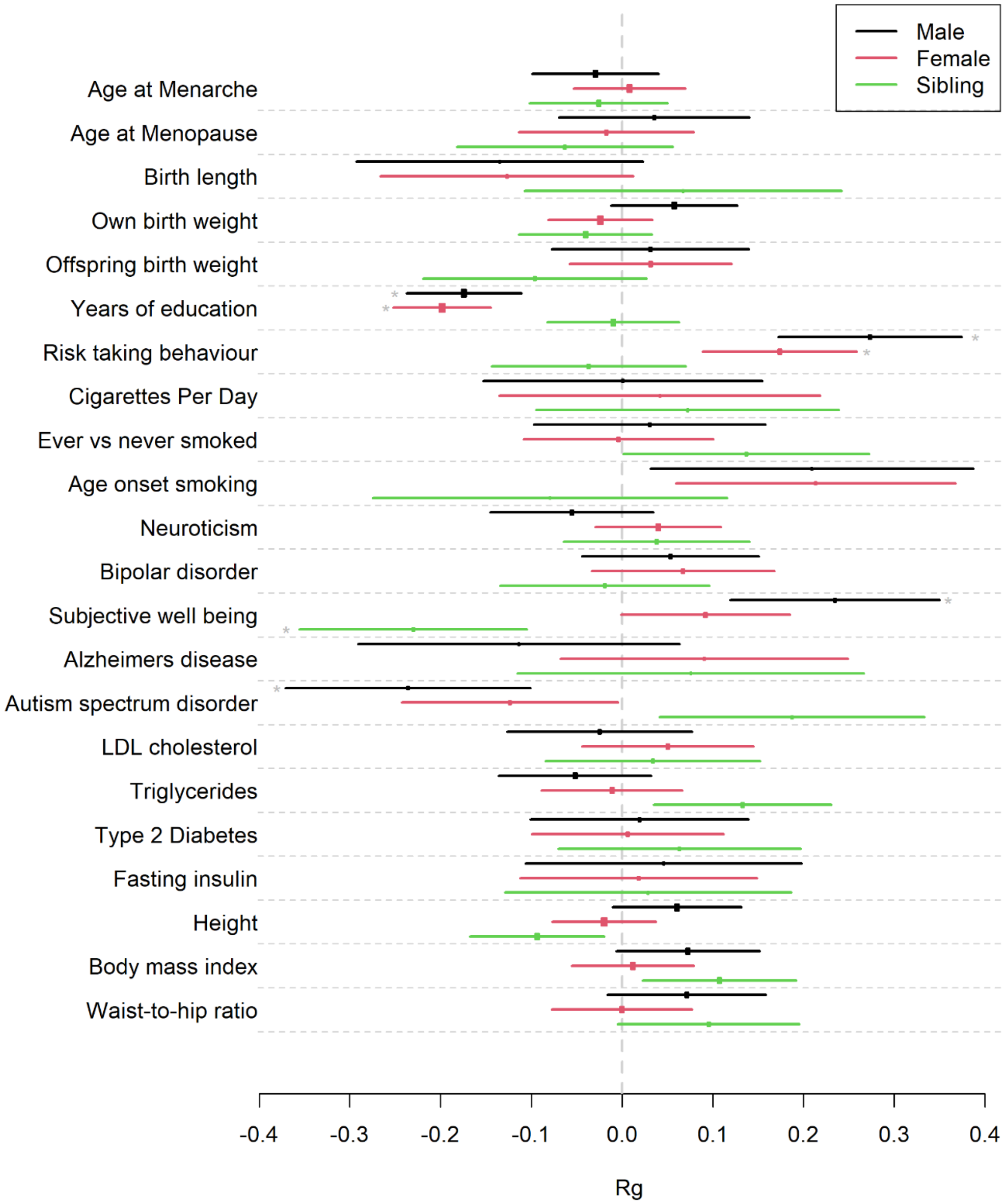
Male
Female
Sibling
Age at Menarche
Age at Menopause
Birth length
Own birth weight
Offspring birth weight
Years of education
Risk taking behaviour
Cigarettes Per Day
Ever vs never smoked
Age onset smoking
Neuroticism
Bipolar disorder
Subjective well being
Alzheimers disease
Autism spectrum disorder
LDL cholesterol
Triglycerides
Type 2 Diabetes
Fasting insulin
Height
Body mass index
Waist-to-hip ratio
-0.4
-0.3
-0.2
-0.1
0.0
0.1
0.2
0.3
0.4
Rg

Supplementary Figure 24: Path diagram illustration of the MTAG model used for estimating maternal (A) and offspring (B) effects on birth weight. The two 'observed variables' (in squares) are the summary results statistics from the genome-wide association study (GWAS) of birth weight of the individual and from the GWAS of the birth weight of their offspring. The latent variables (in circles) are the constructs that we are estimating the genetic effects of. $\beta_{m_{adj}}$ in **A)** and $\beta_{o_{adj}}$ in **B)** path coefficients refer to maternal and offspring effects respectively. The residual genetic variance terms for the birth weight of the individual and their offspring are represented by ϕ_o and ϕ_m respectively. The variance of the SNP is represented by σ^2_{SNP} and variance of the summary results statistics are estimated by σ^2_o and σ^2_{m} for the GWAS of own and offspring birth weight respectively. Finally, c_o and c_m are free parameters for the loadings of own birth weight and offspring birth weight on the latent variable respectively. We note that whilst this underlying model is similar, it is not quite the same as estimating the same parameters as in the SEM.

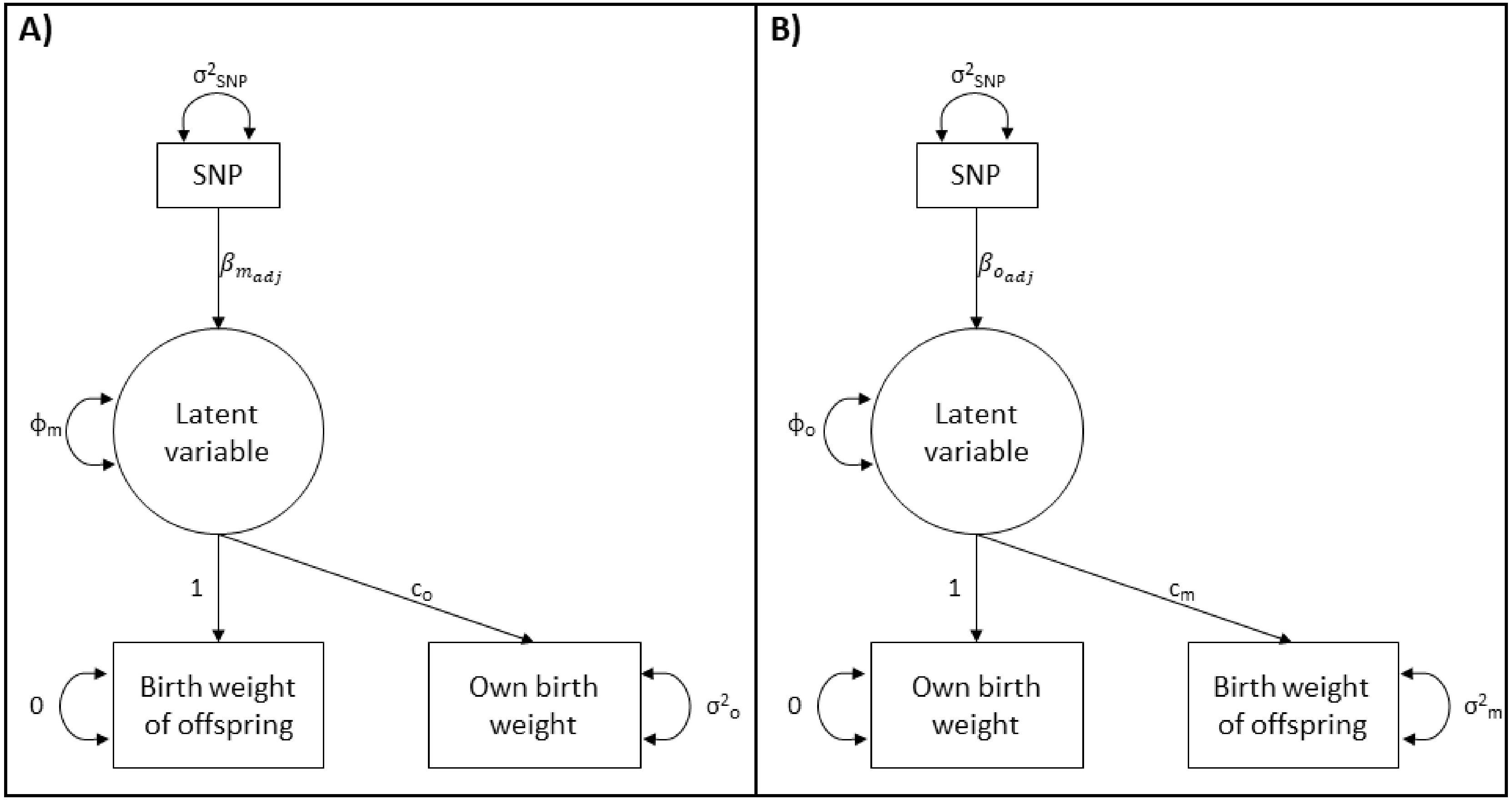
A)
σ2SNP
SNP
βm adj
ϕm
Latent variable
1
co
0
Birth weight of offspring
Own birth weight
σ2o
B)
σ2SNP
SNP
βo adj
ϕo
Latent variable
1
cm
0
Own birth weight
Birth weight of offspring
σ2m

Supplementary Figure 25: Diagram of the structural equation model (SEM) for estimating maternal and offspring genetic effects on birth weight. The three observed variables (in squares) are the birth weight of the individual (BW), the birth weight of their offspring (BW_O) and the genotype of the individual (SNP). The latent variables (in circles) are the genotypes for the individual's mother (G_G) and the genotype of the individual's first offspring (G_O). The total variance of the latent genotypes for the individual's mother (G_G) and offspring (G_O) and for the observed SNP variable is set to Φ (i.e., variance(G_G) = Φ, variance (SNP) = 0.75Φ + 0.25Φ, variance (G_O) = 0.75Φ + 0.25Φ). $\beta_{m_{adj}}$ and $\beta_{o_{adj}}$ path coefficients refer to maternal and offspring effects respectively. The residual error terms for the birth weight of the individual and their offspring are represented by ε and ε_O respectively and we estimate the variance of both of these terms in the SEM. The covariance between residual genetic and environmental sources of variation is given by ρ. A) is used to model the subset of individuals with complete data. B) is used to model the subset of genotyped individuals who report their own birth weight, but not their offspring's birth weight. Genotyped males who report their own birth weight (but not their offspring's) can be incorporated into this part of the model. C) is used to model the subset of genotyped individuals who report their offspring's birth weight, but not their own. These three models are fit to the three subsets of data that contain the various patterns of missingness, and then the likelihoods from each model are combined. Parameter estimates and their standard errors were obtained in OpenMx and the significance of the maternal and offspring effects tested by Wald test.

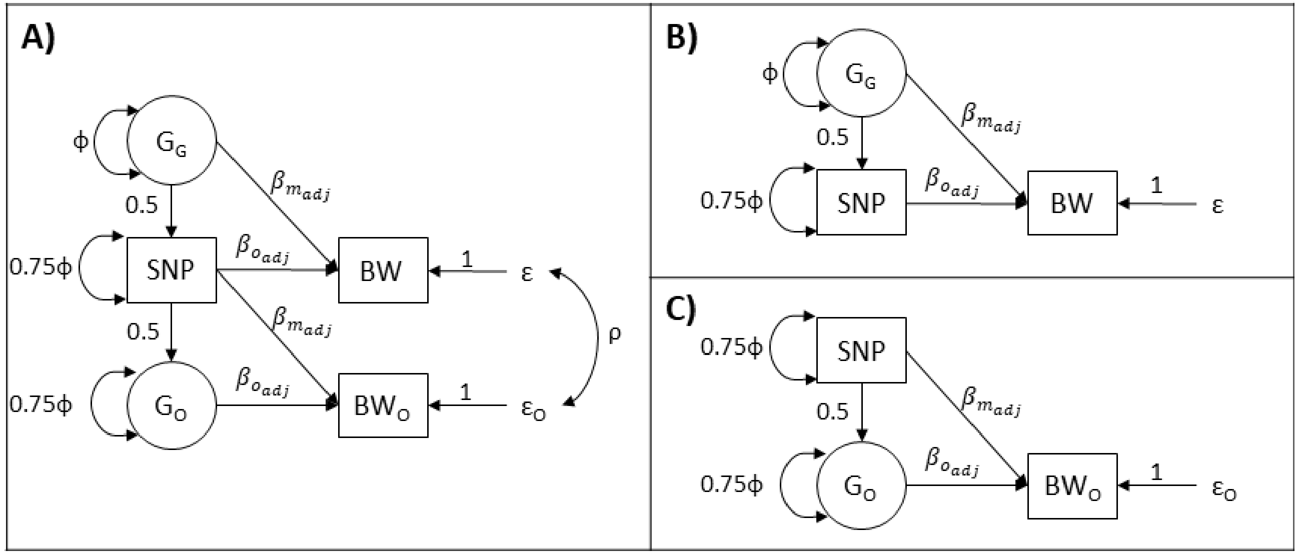

Supplementary Figure 26: Path diagram representation of the mtCOJO model used for estimating maternal (A) and offspring (B) effects on birth weight. mtCOJO is a general framework for estimating the direct effect of a SNP of interest (SNP_i) on an outcome phenotype conditional on one or more covariates, using summary GWAS results data. The basic idea is that the effect of the covariate on the outcome (i.e. β_{Own} in panel A and β_{Off} in panel B) is first estimated using Mendelian randomization analysis i.e. SNPs (SNP_{X1}, SNP_{X2}, SNP_{X3}, SNP_{X4}, SNP_{X5}, SNP_{X6}) that are genome-wide significant from a GWAS of the covariate variable(s) are used as genetic instruments to proxy the covariate variable(s) (as indicated by the path coefficients β_{x1}, β_{x2}, β_{x3}, β_{x4}, β_{x5}, β_{x6}) and estimate the effect of the covariate on the outcome phenotype (i.e. β_{Own} in panel A and β_{Off} in panel B). In the simple case of one covariate (as in this manuscript), the direct effect of the SNP_i on the outcome can then be estimated as the difference between the unadjusted regression of the outcome on SNP_i minus the product of the regression of the covariate on the SNP (β_{ix}) and the effect of the covariate on the outcome (β_{Own}) in panel A and (β_{Off}) in panel B. Within the context of this manuscript, this means that mtCOJO can be used to approximate the direct effect of an individual's own genotype on their offspring' birthweight conditional on their own birthweight (panel A and labelled here as $\beta_{SNP\_off,i}$) or the direct effect of an individual's genotype on their own birthweight conditional on putative effects of their offspring's birthweight (panel B and labelled here as $\beta_{SNP\_own,i}$) We note that whilst this underlying model is similar, it is not quite the same as estimating the same parameters as in the SEM.

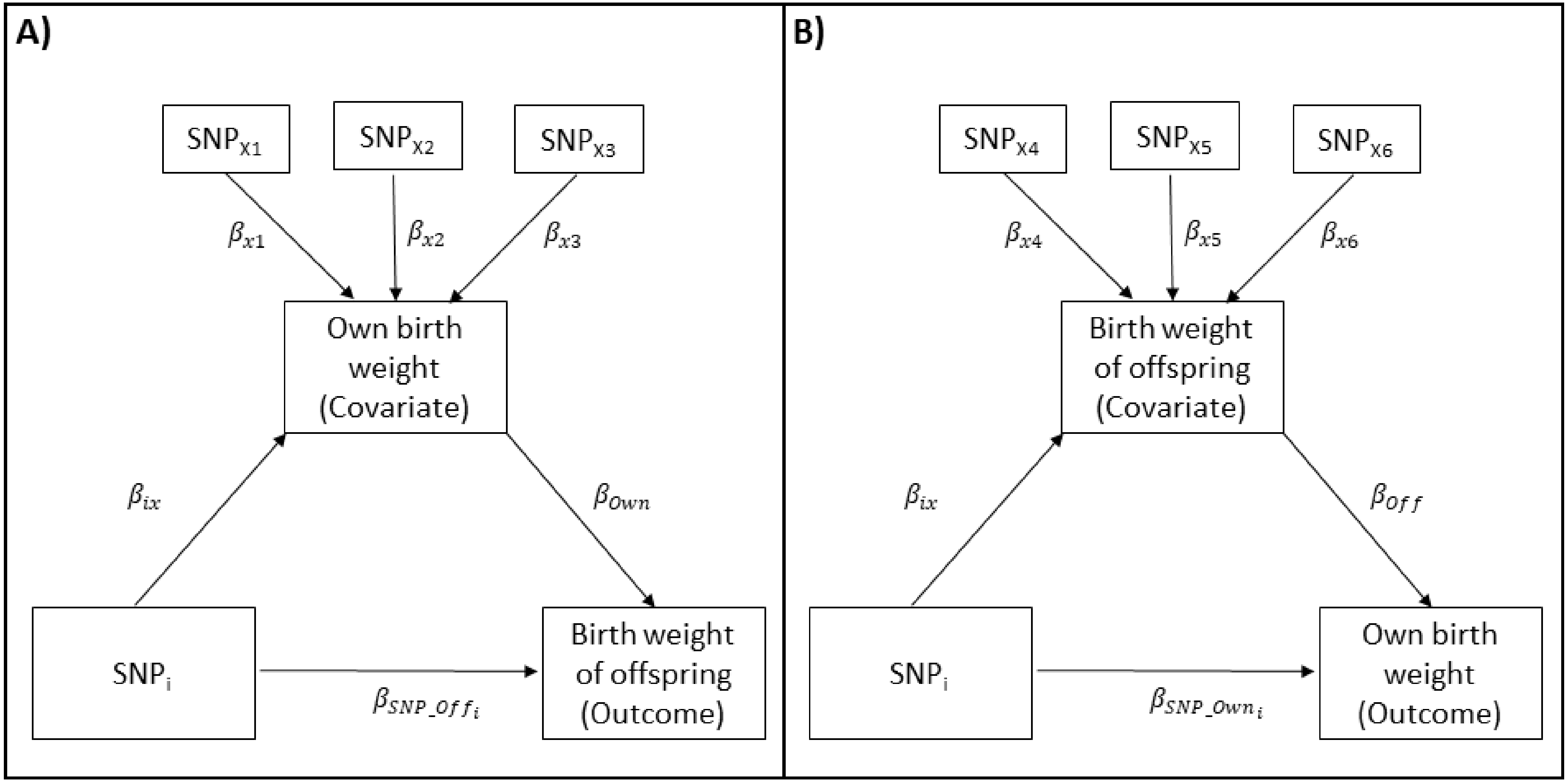
A)
SNP$_{X1}$
SNP$_{X2}$
SNP$_{X3}$
β_{x1}
β_{x2}
β_{x3}
Own birth weight (Covariate)
β_{ix}
β_{Own}
SNP$_i$
$\beta_{SNP\_Off_i}$
Birth weight of offspring (Outcome)
B)
SNP$_{X4}$
SNP$_{X5}$
SNP$_{X6}$
β_{x4}
β_{x5}
β_{x6}
Birth weight of offspring (Covariate)
β_{ix}
β_{Off}
SNP$_i$
$\beta_{SNP\_Own_i}$
Own birth weight (Outcome)

Supplementary Figure 27: Diagram of the genomic structural equation model (Genomic SEM) used for estimating conditional genetic effects on birth weight. The two 'observed variables' (in squares) are the summary results statistics from the genome-wide association study (GWAS) of birth weight of the individual and from the GWAS of the birth weight of their offspring. The latent variables (in circles) are the constructs that we are estimating the genetic effects of. $\beta_{m_{adj}}$ and $\beta_{o_{adj}}$ path coefficients refer to maternal and offspring conditional genetic effects respectively. Genetic variance of the birth weight of the individual and their offspring are represented by ϕ_o and ϕ_m respectively. Finally, ρ represents the genetic covariance between the latent factors, which can be estimated using Genomic SEM (although it was not estimated in our model, indicated by the dotted double arrow).

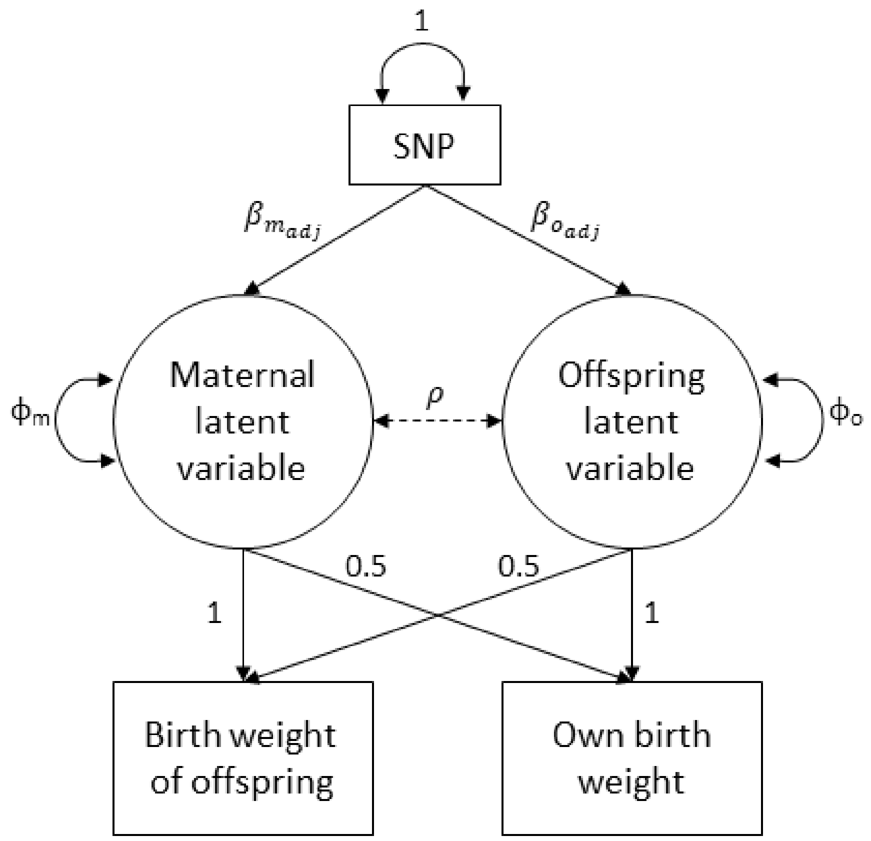

Supplementary Figure 28: Diagram of the genomic structural equation model (Genomic SEM) for estimating conditional genetic effects on fertility. A) is a model that is similar to the birth weight analysis with just the number of siblings and the number of children mothered. B) is the full model incorporating data on the number of children fathered. If desired genetic covariances between the latent genetic factors can be estimated. β_m, β_p and β_s represent conditional maternal, paternal and sibling genetic effects respectively. The symbols ϕ_m, ϕ_p and ϕ_s represent the variance of latent female, male and sibling genetic factors.

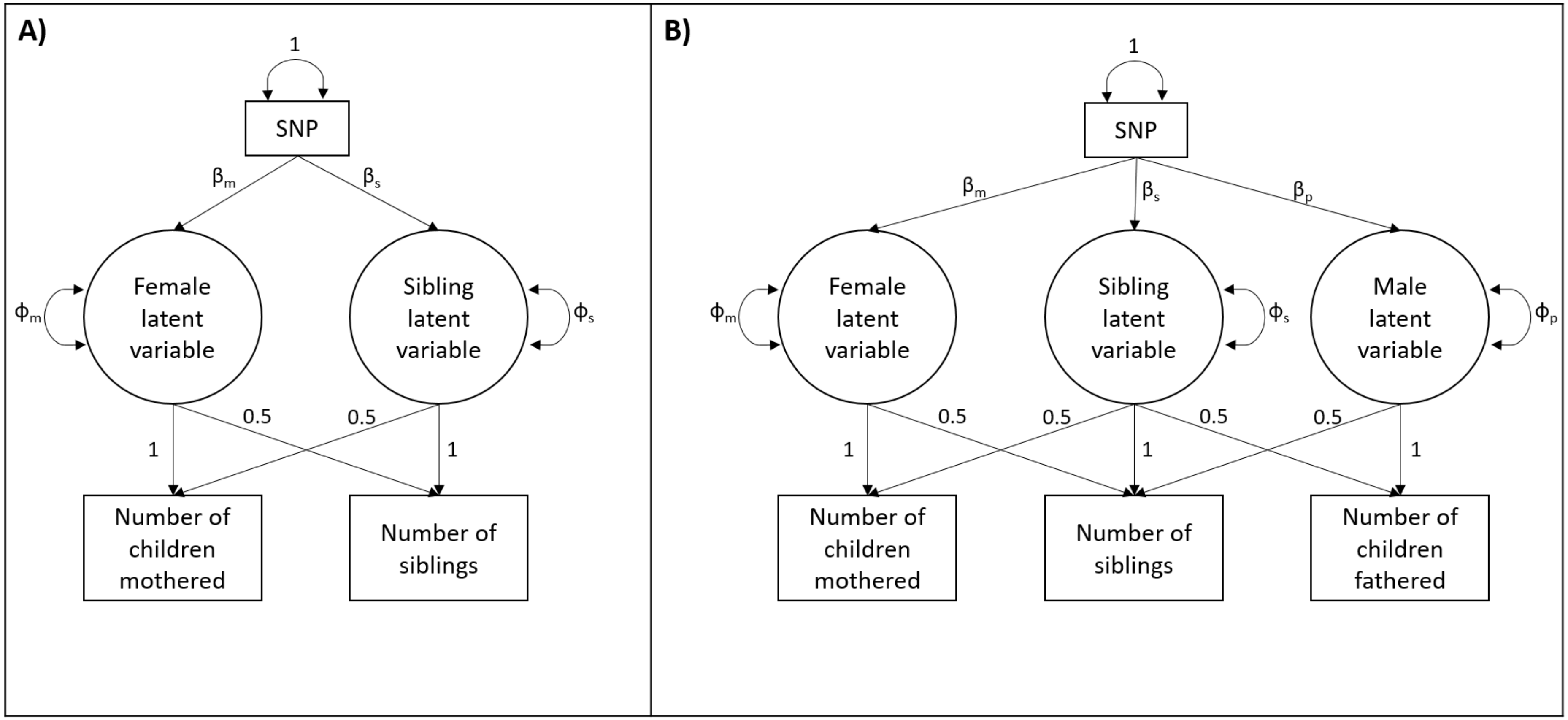

References:

1 Warrington, N. M. *et al.* Maternal and fetal genetic effects on birth weight and their relevance to cardio-metabolic risk factors. *bioRxiv*, 442756, doi:10.1101/442756 (2018).

2 Loh, P. R. *et al.* Efficient Bayesian mixed-model analysis increases association power in large cohorts. *Nat Genet* **47**, 284-290, doi:10.1038/ng.3190 (2015).

3 Warrington, N. M. *et al.* Maternal and fetal genetic effects on birth weight and their relevance to cardio-metabolic risk factors. *Nat Genet* **51**, 804-814, doi:10.1038/s41588-019-0403-1 (2019).

4 Turley, P. *et al.* Multi-trait analysis of genome-wide association summary statistics using MTAG. *Nat Genet* **50**, 229-237, doi:10.1038/s41588-017-0009-4 (2018).

5 Zhu, Z. *et al.* Causal associations between risk factors and common diseases inferred from GWAS summary data. *Nature communications* **9**, 224, doi:10.1038/s41467-017-02317-2 (2018).

6 Grotzinger, A. D. *et al.* Genomic structural equation modelling provides insights into the multivariate genetic architecture of complex traits. *Nature human behaviour* **3**, 513-525, doi:10.1038/s41562-019-0566-x (2019).

7 Bulik-Sullivan, B. *et al.* An atlas of genetic correlations across human diseases and traits. *Nat Genet* **47**, 1236-1241, doi:10.1038/ng.3406 (2015).

8 Zheng, J. *et al.* LD Hub: a centralized database and web interface to perform LD score regression that maximizes the potential of summary level GWAS data for SNP heritability and genetic correlation analysis. *Bioinformatics (Oxford, England)*, doi:10.1093/bioinformatics/btw613 (2016).

9 Barban, N. *et al.* Genome-wide analysis identifies 12 loci influencing human reproductive behavior. *Nature genetics* **48**, 1462-1472, doi:10.1038/ng.3698 (2016).
