# Supplementary material for: Estimating direct and indirect genetic effects on offspring phenotypes using genome-wide summary results data
Source: Nat Commun. 2021 Sep 14;12:5420. doi: 10.1038/s41467-021-25723-z (PMC8440517; doi:10.1038/s41467-021-25723-z)
Supplement: Supplementary file 2 — Description of Additional Supplementary Files [file 41467_2021_25723_MOESM2_ESM.pdf]

Description of Additional Supplementary Files

File Name: Supplementary Data 1
Description: : Comparison of results from each of the methods to the SEM results using individual level data for the 301 autosomal genomewide significant SNPs from the latest GWAS of birth weight when there is sample overlap between the two GWAS.

File Name: Supplementary Data 2
Description: Comparison of results from each of the methods to the SEM results using individual level data for the 301 autosomal genomewide significant SNPs from the latest GWAS of birth weight when there is no sample overlap between the two GWAS.

File Name: Supplementary Data 3
Description: Results from the fertility GWAS for the seven loci that reached genome-wide significance (P < 5x10-8) in the conditional GWAS analysis estimating male, female and sibling specific genetic effects.
