# Supplementary material for: Estimating direct and indirect genetic effects on offspring phenotypes using genome-wide summary results data
Source: Nat Commun. 2021 Sep 14;12:5420. doi: 10.1038/s41467-021-25723-z (PMC8440517; doi:10.1038/s41467-021-25723-z)
Supplement: Supplementary file 7 — Reporting Summary [file 41467_2021_25723_MOESM7_ESM.pdf]

nature portfolio

Corresponding author(s): Nicole Warrington

Last updated by author(s): Aug 4, 2021

Reporting Summary

Nature Portfolio wishes to improve the reproducibility of the work that we publish. This form provides structure for consistency and transparency in reporting. For further information on Nature Portfolio policies, see our Editorial Policies and the Editorial Policy Checklist.

Statistics

For all statistical analyses, confirm that the following items are present in the figure legend, table legend, main text, or Methods section.

n/a	Confirmed	
☐	☒	The exact sample size (*n*) for each experimental group/condition, given as a discrete number and unit of measurement
☐	☒	A statement on whether measurements were taken from distinct samples or whether the same sample was measured repeatedly
☐	☒	The statistical test(s) used AND whether they are one- or two-sided<br>*Only common tests should be described solely by name; describe more complex techniques in the Methods section.*
☐	☒	A description of all covariates tested
☐	☒	A description of any assumptions or corrections, such as tests of normality and adjustment for multiple comparisons
☐	☒	A full description of the statistical parameters including central tendency (e.g. means) or other basic estimates (e.g. regression coefficient) AND variation (e.g. standard deviation) or associated estimates of uncertainty (e.g. confidence intervals)
☐	☒	For null hypothesis testing, the test statistic (e.g. *F*, *t*, *r*) with confidence intervals, effect sizes, degrees of freedom and *P* value noted<br>*Give P values as exact values whenever suitable.*
☒	☐	For Bayesian analysis, information on the choice of priors and Markov chain Monte Carlo settings
☒	☐	For hierarchical and complex designs, identification of the appropriate level for tests and full reporting of outcomes
☐	☒	Estimates of effect sizes (e.g. Cohen's *d*, Pearson's *r*), indicating how they were calculated

Our web collection on statistics for biologists contains articles on many of the points above.

Software and code

Policy information about availability of computer code

Data collection	Data collection was performed centrally by the UK Biobank. No software was used for data collection.
Data analysis	We used BOLT-LMM (v2.3.2), GCTA (v1.90.2), LD score regression (v1.0.0; https://github.com/bulik/ldsc), R (v3.4.3; including OpenMx package v2.6.9), MTAG (including python v2.7.12; https://github.com/JonJala/mtag), plink2 (released 18th March 2019), GCTA (v1.9.2.0beta3, mtCOJO function), GenomicSEM v0.0.2 (installed 9th Jan 2020; https://github.com/GenomicSEM/GenomicSEM) and LDHub (ldsc.broadinstitute.org, v1.9.3) to analyze the data and produce plots for this manuscript. A full description of the software used in this paper is provided in the Methods section, along with references to the relevant journal articles.

For manuscripts utilizing custom algorithms or software that are central to the research but not yet described in published literature, software must be made available to editors and reviewers. We strongly encourage code deposition in a community repository (e.g. GitHub). See the Nature Portfolio guidelines for submitting code & software for further information.

Data

Policy information about availability of data

All manuscripts must include a data availability statement. This statement should provide the following information, where applicable:
- Accession codes, unique identifiers, or web links for publicly available datasets
- A description of any restrictions on data availability
- For clinical datasets or third party data, please ensure that the statement adheres to our policy

Human genotype and phenotype data on which the results of this study were based were accessed from the UK Biobank (http://www.ukbiobank.ac.uk/) with accession ID 53641. The genotype and phenotype data are available upon application from the UK Biobank (http://www.ukbiobank.ac.uk/). GWAS summary statistics from the fertility GWAS are available at the Evans Group website (https://evansgroup.di.uq.edu.au/GWAS_RESULTS/FERTILITY/)upon publication. Genomic

positions are based on NCBI Build 37.

Field-specific reporting

Please select the one below that is the best fit for your research. If you are not sure, read the appropriate sections before making your selection.

- [x] Life sciences
- [ ] Behavioural & social sciences
- [ ] Ecological, evolutionary & environmental sciences

For a reference copy of the document with all sections, see nature.com/documents/nr-reporting-summary-flat.pdf

Life sciences study design

All studies must disclose on these points even when the disclosure is negative.

Sample size	No sample size/power calculations were conducted as the main focus of the manuscript was not to detect novel associations, but rather on comparing association statistics across the different methods. The sample sizes contained within the UK Biobank are large enough for this purpose. Full descriptions of how we defined the samples included in the GWAS of own or offspring birth weight are included in the methods section. We analyzed data from N=183,728 individuals with their own birth weight reported, N= 159,471 who reported their own offspring's birth weight. For the fertility analysis, we included 237,768 women reporting how many children they mothered, 199,570 men reporting how many children they had fathered, and 430,466 individuals reporting how many siblings they have.
Data exclusions	For the analysis of birth weight, we excluded multiple births, individuals whose reported birth weight differed between the follow-ups, individuals with birth weight <2.5 kg or >4.5 kg. Additionally, we excluded individuals who were not of European descent,. For the fertility analysis, we excluded individuals whose response changed over the follow-ups and those who were not of European descent.
Replication	This study focuses on comparing different methods for partitioning genetic effects into parental and offspring components so "replication of findings" does not make sense in this context. No other cohorts large enough to replicate results from the fertility GWAS were available.
Randomization	This study involves a population based cohort and so randomization was not performed.
Blinding	Data collected by the UK Biobank were observational and had no specific interventions. As such, no blinding was required.

Reporting for specific materials, systems and methods

We require information from authors about some types of materials, experimental systems and methods used in many studies. Here, indicate whether each material, system or method listed is relevant to your study. If you are not sure if a list item applies to your research, read the appropriate section before selecting a response.

Materials & experimental systems

n/a	Involved in the study	
x		Antibodies
x		Eukaryotic cell lines
x		Palaeontology and archaeology
x		Animals and other organisms
	x	Human research participants
x		Clinical data
x		Dual use research of concern

Methods

n/a	Involved in the study	
x		ChIP-seq
x		Flow cytometry
x		MRI-based neuroimaging

Human research participants

Policy information about studies involving human research participants

Population characteristics	The UK Biobank is a cohort of British residents between the ages of 37 to 73, recruited to 22 centers at baseline measurement. Of the European-ancestry individuals, who reported their own birth weight, over 60% of participants were women. Analyses of offspring birth weight (maternal GWAS) contained all women; the sex of the baby was not known. Of the European-ancestry individuals who reported the number of children or siblings they had, approximately 50% were female. Full characteristics are presented in the manuscript. Participants were not selected on the basis of disease status.
Recruitment	The UK Biobank consists of participants recruited in middle-age, birth weight data are recalled and self-reported and data on the number of children and siblings were self reported. We note that the UK Biobank had a low response rate (5%), so we cannot rule out potential bias from selection, for example, resulting in higher average socio-economic position. However, any biases should be the same across the different methods tested in this manuscript and so should not affect the interpretation of our results.
Ethics oversight	The UK Biobank has ethical approval from the North West Multi-Centre Research Ethics Committee (MREC), which covers the UK, and all participants provided written informed consent.

Note that full information on the approval of the study protocol must also be provided in the manuscript.
